# Sex role similarity and sexual selection predict male and female song elaboration and dimorphism in fairy‐wrens

**DOI:** 10.1002/ece3.8378

**Published:** 2021-12-07

**Authors:** Karan J. Odom, Kristal E. Cain, Michelle L. Hall, Naomi E. Langmore, Raoul A. Mulder, Sonia Kleindorfer, Jordan Karubian, Lyanne Brouwer, Erik D. Enbody, John Anthony Jones, Jenélle L. Dowling, Ana V. Leitão, Emma I. Greig, Christine Evans, Allison E. Johnson, Kimberley K.‐A. Meyers, Marcelo Araya‐Salas, Michael S. Webster

**Affiliations:** ^1^ Cornell Lab of Ornithology and Department of Neurobiology and Behavior Cornell University Ithaca New York USA; ^2^ Department of Psychology University of Maryland, College Park College Park Maryland USA; ^3^ Research School of Biology Australian National University Canberra ACT Australia; ^4^ School of Biological Sciences University of Auckland Auckland New Zealand; ^5^ School of BioSciences The University of Melbourne Melbourne Vic. Australia; ^6^ Bush Heritage Australia Melbourne Vic. Australia; ^7^ School of Biological Sciences The University of Western Australia Perth WA Australia; ^8^ Max Planck Institute for Ornithology Vogelwarte Radolfzell Germany; ^9^ College of Science and Engineering Flinders University Adelaide SA Australia; ^10^ Department of Behavioural and Cognitive Biology Konrad Lorenz Research Center for Behaviour and Cognition University of Vienna Vienna Austria; ^11^ Department of Ecology and Evolutionary Biology Tulane University New Orleans Louisiana USA; ^12^ Department of Animal Ecology & Physiology Institute for Water and Wetland Research Radboud University Nijmegen The Netherlands; ^13^ Department of Animal Ecology Netherlands Institute of Ecology (NIOO‐KNAW) Wageningen The Netherlands; ^14^ Department of Medical Biochemistry and Microbiology Uppsala University Uppsala Sweden; ^15^ Division of Biological Sciences University of Montana Missoula Montana USA; ^16^ Cornell Lab of Ornithology and Project Feeder Watch Cornell University Ithaca New York USA; ^17^ School of Biological Sciences University of Nebraska‐Lincoln Lincoln Nebraska USA; ^18^ Centro de Investigación en Neurociencias Universidad de Costa Rica San José Costa Rica; ^19^ Esciela de Biología, Universidad de Costa Rica San José Costa Rica

**Keywords:** complexity, dimorphism, life history, *Malurus*, sex roles

## Abstract

Historically, bird song complexity was thought to evolve primarily through sexual selection on males; yet, in many species, both sexes sing and selection pressure on both sexes may be broader. Previous research suggests competition for mates and resources during short, synchronous breeding seasons leads to more elaborate male songs at high, temperate latitudes. Furthermore, we expect male–female song structure and elaboration to be more similar at lower, tropical latitudes, where longer breeding seasons and year‐round territoriality yield similar social selection pressures in both sexes. However, studies seldom take both types of selective pressures and sexes into account. We examined song in both sexes in 15 populations of nine‐fairy‐wren species (Maluridae), a Southern Hemisphere clade with female song. We compared song elaboration (in both sexes) and sexual song dimorphism to latitude and life‐history variables tied to sexual and social selection pressures and sex roles. Our results suggest that song elaboration evolved in part due to sexual competition in males: male songs were longer than female songs in populations with low male survival and less male provisioning. Also, female songs evolved independently of male songs: female songs were slower paced than male songs, although only in less synchronously breeding populations. We also found male and female songs were more similar when parental care was more equal and when male survival was high, which provides strong evidence that sex role similarity correlates with male–female song similarity. Contrary to Northern Hemisphere latitudinal patterns, male and female songs were more similar at higher, temperate latitudes. These results suggest that selection on song can be sex specific, with male song elaboration favored in contexts with stronger sexual selection. At the same time, selection pressures associated with sex role similarity appear to favor sex role similarity in song structure.

## Graphical abstract

Birdsong is often studied as a sexually selected male trait; yet, in many species, both sexes sing. Here, we examined selection pressures responsible for song elaboration and sexual dimorphism in 15 populations of 9 fairy‐wren species (Maluridae), an Australasian lineage with female song. We provide evidence that sex role similarity and sexual selection contribute to male–female song similarity and dimorphism in this group. These results suggest that selection on song can be sex specific, with male song elaboration favored in contexts coincident with sexual selection, while selection pressures associated with sex role similarity also appear to constrain sex‐specific song evolution and song dimorphism.

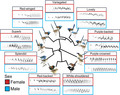

## 1 INTRODUCTION

Many elaborate traits, including conspicuous colors, complex vocalizations, and vigorous displays, that have traditionally been thought of as sexually selected in males are also expressed in females and can serve broader functions (Amundsen, 2000; Dale et al., 2015; Langmore, 1998; Odom et al., 2014). These findings suggest a more complex selective landscape responsible for trait elaboration (Price, 2015; Rosvall, 2011; Tobias et al., 2012). Sexual selection theory makes straightforward predictions that traits should be more elaborate when competition for mating is high (Andersson, 1994; Tobias et al., 2012). This theory is less applicable to females, as access to mates is seldom what affects variance in female reproductive success (Emlen & Oring, 1977; West‐Eberhard, 1983; Rubenstein & Lovette, 2009, but see Langmore et al., 1996). Instead, evidence suggests that elaborate female traits often function in female–female competition to gain access to resources for themselves or their offspring, and less often exclusively to obtain mates (Rosvall, 2011; Tobias et al., 2012). Thus, social selection in the form of intra‐sexual competition for resources other than mates may have played a particularly large role in the evolution of elaborate female traits (Clutton‐Brock, 2009; Rosvall, 2011; Tobias et al., 2012).

Similarly, sexual dimorphism is thought to evolve primarily through strong sexual selection for elaboration in males (Andersson, 1994; Catchpole, 1982). However, dimorphism may arise from a number of selective pressures, including initial selection for elaborate traits in both sexes, followed by selection away from elaboration in females (Dale et al., 2015; Hofmann et al., 2008; Odom et al., 2014). Along these lines, recent research shows that elaborate female traits can be selected against by nest predation risk or other reproductive costs associated with those traits (Soler & Moreno, 2012; Kleindorfer et al., 2016; but see Cain et al., 2019). In such instances, multiple selective pressures acting in concert on both males and females could also influence overall patterns of sexual dimorphism (Dale et al., 2015; Hofmann et al., 2008; Johnson et al., 2013; Wiens, 2001). In other instances, male and female elaborate traits can be selected for by similar selective pressures, but the strength or direction of selection may differ between the sexes (Dale et al., 2015; Soler & Moreno, 2012). Therefore, to understand how dimorphism evolves, it is important to consider the selective pressures that promote similarity, as well as differences, and consider how those pressures shape both males and females (Price, 2015).

Bird songs offer an excellent system to investigate the range of selective pressures that have shaped variation in male and female elaboration and dimorphism. Northern temperate male bird song is thought to be sexually selected, but other forms of selection have attracted less attention (Catchpole & Slater, 2008). The recent realization that female bird song is globally widespread and ancestral suggests that diverse selective pressures are likely to act on females, as well as males, to produce the range of variation in male and female songs seen across species today (Odom et al., 2014; Riebel et al., 2019).

One proposed pattern commonly associated with sexual selection on male song is increased song complexity at higher, more temperate latitudes where there may be more divergent sex roles (Najar & Benedict, 2019; Read & Weary, 1992; Catchpole & Slater, 2008). Specifically, it is hypothesized that increased complexity at higher latitudes occurs because of increased sexual selection on migrating males to establish territories quickly and attract females during short, synchronous temperate breeding seasons (Catchpole, 1982; Slater & Mann, 2004). Therefore, latitude is thought of as a proxy for the variation in life‐history patterns observed across tropical to temperate environments (e.g., Martin, 1996). However, support for an association between latitude and song complexity is mixed. About half of the studies on this topic find no support for this or even the opposite pattern (reviewed in Najar & Benedict, 2019). Variation in these findings could be due to which song metrics are compared within each of these studies (Benedict & Najar, 2019) to different evolutionary pressures acting on different aspects of song structure (e.g., sexual selective pressures vs. morphological constraints; Cardoso & Hu, 2011; Derryberry et al., 2012; Greig et al., 2013), or to a lack of association between latitude and the expected life‐history traits in the species examined. However, virtually all these studies have focused on northern temperate or Palearctic species. Latitudinal patterns of avian life history may differ in the equatorial regions and Southern Hemisphere from those observed in the Northern Hemisphere (Martin, 1996; Robinson, 1949). Specifically, with fewer migratory species in the Southern Hemisphere, we may expect to find life‐history patterns there that are more similar to those seen in the tropics (Lloyd et al., 2014; Samaš et al., 2013). Therefore, the Southern Hemisphere offers a good system to directly investigate relationships between song structure and life history in a part of the world where life history may not covary as strongly with latitude. In general, biogeographic patterns of song and life history in the Southern Hemisphere are a much needed area of study (Martin, 1996; Xiao et al., 2017).

In addition, to decipher overall patterns of bird song complexity, we need to incorporate life‐history patterns and selective pressures relevant to both females and males (Price, 2015; Riebel et al., 2019). At low, tropical latitudes avian life histories associated with more sedentary lifestyles seem to select for more similar sex roles. The logic is as follows: at lower tropical latitudes, pairs often defend a territory year‐round, are longer lived, and may breed for extended parts of the year (Martin, 2015; Stutchbury & Morton, 2001; Tobias et al., 2016). Longer lifespans coupled with year‐round territoriality and partnerships probably result in fewer vacant territories and potential partners, and thus higher levels of competition for breeding opportunities (Slater & Mann, 2004). In addition, nest predation rates are higher in the tropics when controlling for nesting period, so, along with longer breeding seasons, this may necessitate greater bi‐parental care (Freeman et al., 2019). Altogether, these life‐history patterns appear to select for suites of traits, including socially monogamous, long‐term pair bonds, and similarity in sex roles in many non‐migratory songbirds (Slater & Mann, 2004). Indeed, several studies find correlations between the presence of female song or male–female duets and year‐round territoriality, monogamy, long‐term pair bonds, and tropical or sedentary life histories (Benedict, 2008; Logue & Hall, 2014; Price, 2009; Tobias et al., 2016). Therefore, the selective pressures that mediate sex role similarity in tropical regions may also influence the evolution of ornamental traits in both females and males, selecting for more similar behaviors and similar levels of trait elaboration and should be incorporated into studies of avian song complexity (Slater & Mann, 2004).

In order to investigate these biogeographical patterns and the potential selective pressures that may have shaped male and female variation in song, we compared a suite of life‐history traits with elaboration and dimorphism of male and female songs within and among the nine fairy‐wren species belonging to the Maluridae, a well‐studied clade with extensive breeding, social organization, and life‐history data (Brouwer et al., 2017). We addressed three main questions: (1) how similar are male and female songs within and among species? (2) which life‐history traits are associated with elaboration in male and female songs? and (3) which life‐history traits are associated with dimorphism between male and female songs? We examined song elaboration in males and females separately from song dimorphism in order to assess selective pressures that may be acting on male and female songs independently (Price, 2015). To evaluate possible selective pressures associated with song elaboration and dimorphism, we compared a number of reproductive and social variables to song structure, including extra‐pair paternity, brood size, breeding synchrony, proportion of provisioning by males, adult male survival, group size, breeding density, and latitude.

If male and female song elaboration has been sexually selected, then we expect song elaboration in each sex to increase with aspects of fairy‐wren breeding behavior that are associated with competition for mates, such as extra‐pair mating, brood size, and breeding synchrony. Extra‐pair paternity is likely to reflect male variability in reproductive success (Brouwer & Griffith, 2019; Macedo et al., 2008). Similarly, breeding synchrony may represent increased competition for social and extra‐pair mates at the start of the breeding season, whereas brood size may reflect variation in female reproductive investment (Catchpole & Slater, 2008; Stutchbury & Morton, 2001). Conversely, if male and female song structure has been socially selected or has been shaped more exclusively by intra‐sexual social competition, then we expect elaboration in each sex, and dimorphism, to increase with aspects of social structure, such as group size or breeding density per population. Lastly, if song elaboration and dimorphism are influenced by selective pressures associated with sex role similarity, then we expect male and female songs to be most similar in conditions in which male and female roles are similar (e.g., high rates of bi‐parental care, pair‐breeding (rather than cooperative breeding) and potentially at low, tropical latitudes). Moreover, male songs may be comparatively more elaborate when sex roles are more divergent (e.g., low rates of paternal care and in temperate regions). These hypotheses are not mutually exclusive, but rather may work together to shape overall patterns of female and male variation in song.

## 2 METHODS

In order to examine factors influencing sex differences in song, we obtained recordings and life‐history data for 15 populations of 9 species of fairy‐wrens, resulting in data for the following species and populations in Australia and Papua New Guinea: lovely (*Malurus amabilis*; Cairns, QLD, 16.9186°S, 145.7781°E), purple‐backed (*M*. *assimilis*; Brookfield Conservation Park, SA, 34.3615°S, 139.4862°E (*M. a*. *assimilis*) & Kakadu, NT (*M. a*. *dulcis*) 13.43°S, 132.42°E), purple‐crowned (*M*. *coronatus*; Mornington, WA, 17.5289°S, 126.1034°E), red‐backed (*M*. *melanocephalus*; Herberton, QLD, 17.3833°S, 145.3833°E, & Lake Samsonvale, QLD, 27.2613°S, 152.9000°E), red‐winged (*M*. *elegans*; Smith Brook, WA, 34.3658°S, 116.2072°E), splendid (*M*. *splendens*; Brookfield Conservation Park, SA, 34.3615°S, 139.4862°E), superb (*M*. *cyaneus*; Australian National Botanic Gardens, ACT, 35.2789°S, 149.1089°E, Campbell Park, ACT, 35.2822°S, 149.1722°E, Cleland, SA, 34.9701°S, 138.6941°E, Kangaroo Island, SA 35.7752°S, 137.2142°E, Lara, VIC, 38.00°S, 144.41°E), variegated (*M*. *lamberti*; Lake Samsonvale, QLD, 27.2613°S, 152.9000°E), and white‐shouldered (*M. alboscapulatus lorentzii*; Obo, Western Province, New Guinea, 7.6056°S, 141.3064°). See Figure S1 for a map of the sampling locations.

### 2.1 Study species

Fairy‐wren species share many aspects of their natural history (all species are insectivorous, sedentary, socially monogamous), but populations and species differ in important aspects of their ecology, including levels of extra‐pair paternity, breeding season length, and cooperative breeding (with different proportions of individuals delaying dispersal to help raise young). These behaviors have been well‐studied across populations of several fairy‐wren species and were recently collated into a single dataset including reproductive rates, breeding densities, parental care, and survival (Brouwer et al., 2017). Fairy‐wrens are also a good study system for this investigation because both males and females regularly sing (Evans & Kleindorfer, 2016; Mahr et al., 2016; Rowley & Russell, 1997). Male and female songs have been well‐studied in certain species, providing evidence that male and female songs play a role in territory defense and same‐sex competition (Cain & Langmore, 2015; Colombelli‐Négrel, 2016; Cooney & Cockburn, 1995; Dalziell & Cockburn, 2008; Dowling & Webster, 2013; Hall & Peters, 2008; Kleindorfer et al., 2013), and female song is related to annual reproductive success and habitat quality (Cain et al., 2015; Cain & Langmore, 2016). Male fairy‐wrens of many species give two song types: type I and type II songs (Langmore & Mulder, 1992); whereas females largely only give type I songs (Langmore & Mulder, 1992; Rowley & Russell, 1997; but see Greig & Pruett‐Jones, 2008). Type I songs are frequently produced throughout the day by both sexes and by males at dawn and appear to be used in territory defense, whereas male type II songs are delivered either in the dawn chorus or during the day immediately following a loud sound, such as the call of a predatory bird (Dalziell & Cockburn, 2008; Greig & Pruett‐Jones, 2010; Greig & Webster, 2014; Langmore & Mulder, 1992). Comparative studies across fairy‐wrens suggest that male type I song structure is shaped by sexual selection, as well as other selective pressures (Greig et al., 2013). In the current study, we compare the structure of male and female type I songs.

### 2.2 Field recordings

Most recordings for this study were collected by K. Cain in 2015 and 2016 (Austral summer) with recordings for specific, additional populations contributed by individual researchers or research groups studying those populations. All recordings were collected from banded populations of birds and most recordings were collected from free‐roaming birds during natural singing bouts or following experimental playback trials. The stimuli varied depending on the research focus within the population the recordings came from, most being natural songs of male and/or female conspecifics. Recordings of lovely fairy‐wrens were supplemented with recordings of wild birds temporarily caged during experimental trials because these recordings were of higher quality than the natural recordings available for this species (Leitão et al., 2019). Table S1 contains a full list of sample sizes for males and females at each recording location, including details about dates, recording equipment, and playback stimuli used.

### 2.3 Life‐history data

Life‐history data were compiled primarily from Brouwer et al. (2017). Where we had recordings for additional populations, we extended the life‐history dataset. This included adding the following populations to the dataset using existing breeding season data: Campbell Park, Kakadu, Kangaroo Island, Cleland, and Cairns (Colombelli‐Négrel et al., 2009; Leitão et al., 2019, 2021). We also added additional years of data to the original Brouwer dataset for Western Province, New Guinea (Enbody et al., 2019). Variables used from Brouwer et al. (2017) included population‐specific estimates of proportion of extra‐pair offspring, brood size, breeding synchrony, group size, density of breeding males in the population, average proportion of provisioning nest visits made by the dominant male in pairs without helpers, latitude of the field site, and mean annual adult male survival (an estimate of male survival that may reflect variation in both male and species survival rates per population). See Brouwer et al. (2017) for details on how each variable was calculated at the population level. We evaluated possible correlations among these variables. We identified collinearity among explanatory variables (*r* > .7; Dormann et al., 2013) using a Pearson product–moment correlation test and excluded collinear variables. Correlations among most explanatory variables were well below a correlation coefficient of 0.50 (see Table S2).

### 2.4 Phylogenetic tree

For our phylogenetically controlled mixed models, we used overall topology from the most recent species tree for the fairy‐wrens (Lee et al., 2012). This well‐resolved tree is based on 18 molecular markers, including exons, introns, and mitochondrial DNA. We used Mesquite v3.6 to manually place purple‐backed fairy‐wren (*M. assimilis*), a recent species split from the variegated fairy‐wren (*M. lamberti*), as sister to the lovely fairy‐wren (*M. amabilis*). This position for the added branch is justified by multiple findings that purple‐backed fairy‐wrens are sisters to lovely fairy‐wrens (Driskell et al., 2011; McLean et al., 2012). We then calibrated this uncalibrated version of the Lee et al. (2012) tree. We used the chronos function in the ape package in R (Paradis et al., 2004; R Core Team, 2015) to assign nodes in common with a recent, well‐resolved supermatrix phylogeny for the Meliphagides by Marki et al. (2017) as hard values. From these values, we estimated branch lengths and values for nodes not shared in both trees. The resulting tree only included species‐level relationships, but includes the most up‐to‐date information on species‐level relationships and divergence estimates within this clade.

### 2.5 Song analysis

We gathered fine‐structural measurements for all elements in every song. We defined an element as a single, continuous trace on a spectrogram, separated from other elements by a visible break in time. We compiled the measurements into element‐level and song‐level datasets. This allowed us to incorporate both element‐level structure and overall song structure into our analyses. The element‐level acoustic data were used to estimate element diversity, which was added as a single metric to the song‐level dataset. We then used the song‐level data to evaluate sex differences in song structure, song elaboration, and song dimorphism across species. Here and throughout the rest of the paper, we use the term “elaborate” to describe acoustic variables or suites of acoustic variables that are typically associated with song complexity or output and that vary directionally such that larger quantities are considered “more elaborate.” In our analyses, these variables included song duration, element diversity, element number, frequency range, and element rate. As these measures, or combinations of these measures of elaboration could be redundant or under different selection pressures, we combined them into statistically relevant or distinct axes, using principal component analysis (PCA), resulting in three elaboration (PC) scores (as explained in the *Song elaboration* subsection of the statistical analyses below).

All songs were recorded in uncompressed WAV format and all recordings were standardized to a sample rate of 44.1 kHz and a bit depth of 16 prior to measuring. We used Raven Sound Analysis Software v1.5 (Bioacoustics Research Program, 2017) to select and extract measurements for the fundamental frequency for each element in each song, excluding additional harmonics as much as possible. Only high signal‐to‐noise songs were used in which all notes of the song could be readily deciphered from background noise and with no overlap by non‐target species or individuals. Measurements were made by three research technicians and KJO, and all measurers were trained by KJO using a single written protocol. Furthermore, all measurements were extensively checked during and after measuring by KJO both visually and via data exploration for outliers. For spectrographic analysis we used a 512‐point window length with a 90% window overlap and a Hann window function for a time resolution of 1.161 ms and a frequency resolution of 86.1 Hz.

#### 2.5.1 Element‐level acoustic parameters

For the element‐level acoustic space, for every element in all songs we extracted robust, energy‐based measurements of time and frequency from Raven v1.5 (Bioacoustics Research Program, 2017). We then transferred all selections to R using the Rraven package (Araya‐Salas, 2017) and extracted additional acoustic parameters using the R warbleR package (Araya‐Salas & Smith‐Vidaurre, 2017). Prior to random forest analysis (see below), we removed highly correlated acoustic variables (*r* ≥ |.95|), resulting in the following sets of parameters extracted from each program and used in subsequent analysis: Raven—duration 90%, interquartile range duration, center time, frequency 5%, frequency 25%, frequency 75%, 90% bandwidth, interquartile bandwidth, peak frequency, maximum of peak frequency contour, range of the peak frequency contour, slope of peak frequency contour, number of inflections in peak frequency contour; warbleR—mean frequency, standard deviation of frequency, median frequency, skewness, time entropy, entropy, spectral flatness, modulation index, and dominant frequency slope. For a few instances of the slope of peak frequency contour metric, elements were too short to correctly calculate a value. We filled these missing measurements with zero. This is a fair representation as these elements were too short to show any significant frequency modulation. Robust measurements of frequency and time were calculated in Raven from the cumulative amplitude of the spectrum or envelope, as applicable (Charif et al., 2003). See Table S3 and Raven or warbleR manuals for detailed definitions of each parameter (Araya‐Salas & Smith‐Vidaurre, 2017; Charif et al., 2003).

#### 2.5.2 Song‐level acoustic parameters

The following seven acoustic parameters were extracted from the elements composing each song and used in subsequent statistical analyses: song duration (difference between the start and end times of the song), frequency 5% and 95%, frequency range (range between top and bottom frequency among all elements), element number (the total number of elements per song), element rate (element number divided by song duration), and element diversity (a 95% minimum convex polygon area calculated from an element‐level acoustic space, see *Estimation of element diversity* section). Frequency 5% and 95% were not used in analyses involving elaboration since higher or lower frequencies on their own are not considered more or less elaborate, but these parameters were used in all other analyses. We assessed the extent of dimorphism based on male–female differences in any acoustic feature or combination of features. In this way, we also evaluated dimorphism in acoustic variability (the difference in magnitude of variation between male and female songs in acoustic feature space).

### 2.6 Statistical analysis

We evaluated differences in male and female song structure across fairy‐wrens by comparing correct classification of male and female songs in acoustic space using random forest models. In line with our goals, we then used phylogenetically informed mixed models to assess statistically: (1) species and sex differences in song, (2) correlations between song elaboration in both sexes and the life‐history variables, and (3) correlations between sexual dimorphism in song and the life‐history variables. Multiple steps were involved in creation of acoustic spaces and the variables used in each analysis differed slightly. We primarily used a combination of supervised and unsupervised random forest techniques to plot and differentiate among male and female songs in acoustic space. Random forest is a machine learning approach for, that is particularly well‐suited, classification and regression based on constructing and evaluating a large number of decision trees. Supervised random forest uses labeled data to assess the correct assignment of data to a particular category. In unsupervised random forest, the data are unlabeled and classification or exploring patterns within the data is often the goal (Ramasubramanian & Singh, 2016). The steps, metrics, and analyses used in each analysis are explained below.

#### 2.6.1 Species and sex differences in song

To evaluate species and sex differences in song, we used supervised random forests to assess correct classification of male and female songs. We then used Bayesian phylogenetic mixed models to assess statistically which song variables contributed to sex differences in each species.

We calculated correct classification of male and female songs on a species‐by‐species basis within a song‐level acoustic space. For this analysis, we calculated separate acoustic spaces for each species in order to have the highest resolution to test discrimination between male and female songs. We used all seven song‐level acoustic parameters in each species’ acoustic space. To create the acoustic spaces, we conducted a supervised random forest analysis in the R package ranger (Wright & Ziegler, 2017) with the following parameters: 10,000 trees, minimal node size of 1, Gini impurity index as split rule, and three randomly sampled variables at each split. We used the out‐of‐bag error as an indicator of classification accuracy for each species. Out‐of‐bag error is measured as the classification of each sample with a model that was trained without that particular sample. Phylogenetic comparisons for all analyses are described below.

#### 2.6.2 Song elaboration

To evaluate whether song elaboration is correlated with any of the life‐history variables, we created a reduced set of elaboration variables using principal component analysis (PCA). PCA was conducted using the princomp function in R (R Core Team, 2015) on the correlation matrix with the following song‐level acoustic parameters: song duration, frequency range, element number, element rate, and element diversity. Frequency 5% and 95% related parameters were not used in this analysis, as there are no distinct predictions about how these frequency metrics represent song elaboration. We extracted PC scores for the first three principal components to be used in phylogenetic comparative analyses.

#### 2.6.3 Song dimorphism

To evaluate whether song dimorphism is correlated with any of the life‐history variables, we created a reduced set of song dimorphism variables to examine the life‐history variables in phylogenetically informed mixed models. We computed three metrics representing male–female song dimorphism based on male–female differences in a song‐level acoustic space that contained all species: 1. Male–female acoustic area overlap, 2. Male–female acoustic area distance, and 3. Male–female acoustic area size difference. These three dimorphism metrics captured a range of ways that male and female songs can vary in acoustic space. The song‐level acoustic space was created from unsupervised random forests in the R package randomForest (Liaw & Wiener, 2002) using all song‐level acoustic parameters as input variables and using the following function parameter settings: 10,000 trees, minimal node size of 1, Gini impurity index as split rule, three randomly sampled variables at each split, and out‐of‐bag proximity. The resulting proximity matrix was transformed into a set of two vectors by Kruskal's Non‐metric Multidimensional Scaling using the function isoMDS in the R package MASS (Venables & Ripley, 2002). We then computed 95% minimum convex polygon areas for each sex within each population to represent an acoustic area.

We used these acoustic areas to calculate the three male–female song dimorphism metrics. Male–female acoustic area overlap was calculated by population as the area overlap of male and female 95% minimum convex polygons, taken as a proportion of the entire acoustic area for each population. Male–female acoustic area distance was calculated as the distance between the centroids of the male and female acoustic areas. Centroids were calculated as the average values for each dimension of the acoustic space and the distance between centroids was calculated using the dist function in R. Dimensions of the acoustic space (i.e., MDS vectors) were z‐transformed to make male–female centroid distances comparable across populations and species. Male–female acoustic area size difference was calculated as the absolute value of the difference between male and female acoustic areas, proportional to the total acoustic area of the population. This value represents the size difference between male and female songs in acoustic space, or rather, differences in male–female song variability. To control for sample size differences between the sexes when computing each dimorphism metric, we selected a random subset of the data equal to the sample size of the sex with fewer samples. We did this over 100 iterations and then averaged the resulting dimorphism values.

#### 2.6.4 Estimation of element diversity

We calculated element diversity for each song as the 95% minimum convex polygon area surrounding that song's elements in an element‐level acoustic space, using the function mcp in the adehabitatHR R package (Calenge, 2015). Therefore, each area serves as an estimate of the variability or diversity of elements for a given song among all other song elements in the acoustic space. The resulting element diversity values were used in the above song‐level analyses. For comparisons across all species, we created the element‐level acoustic space by inputting all element‐level acoustic parameters for every element for all songs of all species into an unsupervised random forest using the randomForest package in R (Liaw & Wiener, 2002). Similarly, we created species‐specific element diversity scores for our evaluation of correct classification of males and females to their respective sex, which was evaluated by species. For the creation of both sets of element diversity scores, the random forest was run with the following specifications: 10,000 trees, minimal node size of 1, Gini impurity index as split rule, five randomly sampled variables at each split, and out‐of‐bag proximity. This produced a proximity matrix, which we transformed into a set of five vectors using classic multidimensional scaling (MDS). MDS was performed using the cmdscale function in the stats R package. We used the first two MDS vectors to create at 2‐D acoustic space containing all elements of all songs. We then extracted 95% minimum convex polygon areas defined by the elements for each song using the function mcp in the adehabitatHR R package (Calenge, 2015). Therefore, each area quantitatively represented element diversity for each song in subsequent analyses.

#### 2.6.5 Phylogenetic mixed models

We constructed Bayesian phylogenetically controlled mixed models with MCMCglmm in R (Hadfield, 2010) to evaluate the extent of male and female song differences statistically and to evaluate whether life‐history variables correlate to song elaboration or dimorphism among fairy‐wren populations. For models investigating sex differences in song, we ran separate models for each song‐level acoustic parameter as the response variable. Each model controlled for phylogeny, contained sex, species, and their interaction as fixed effects, and individual ID and population nested within species as random intercepts to account for non‐independence of songs from the same bird and birds from the same population.

We also ran separate univariate models for each life‐history trait; as each trait had different instances of missing values and the life‐history variables were averaged per population. This approach prevented model overfitting. We ran six sets of models: one for each of the three PC elaboration scores and one for each of three dimorphism metrics as response variables. Each life‐history variable was included as a fixed effect in each univariate model, and to evaluate potential sex differences in response to life history, for models of song elaboration, we also included sex and its interaction with each life‐history trait as fixed effects. Individual ID, species, and population nested within species were included as random intercepts. These analyses allowed us to assess if song elaboration and dimorphism were correlated to any life‐history traits across all 15 fairy‐wren populations.

For all models we used a non‐informative, parameter‐extended prior to improve mixing with R‐structure V = 1, nu = 0.002 and G‐structure V = 1, nu = 1, alpha.mu = 0, and alpha.V = 25^2, however, overall results did not differ with prior specification. Models of male–female song differences were run with 1,750,000 iterations, a burn‐in of 300, and thinning of 15. Life‐history models for elaboration were run with 300,000 iterations, a burn‐in of 300, and thinning of 30. Life‐history models for dimorphism were run with 500,000 iterations, a burn‐in of 500, and thinning of 30. We visually examined model diagnostics for all results to ensure stationarity had been reached and computed levels of autocorrelation which were <0.1 for all parameters (supplementary material—model diagnostics). We used Bayesian *p*‐values, a value analogous to traditional *p*‐values, with a cutoff of less than or equal to 0.05 to evaluate which variables contributed substantially to variation in each model.

In addition, we also performed model selection procedures for multivariate comparative analyses of elaboration and dimorphism compared to life‐history traits using the function dredge in the R package MuMIn (Barto´n & Kamil, 2019). We used the same priors, burn‐in, thinning, and number of iterations as for the univariate analyses. To compensate for explanatory variables with missing data, we scaled all explanatory variables and set the missing values to zero, that is, the mean (Nakagawa & Freckleton, 2011). Selection of the best model was determined based on DIC. If top models differed by less than two DIC values, then we chose the model with the fewest parameters. The appropriateness of using DIC for model selection is debated (Spiegelhalter et al., 2014). Therefore, we used the model selection results mainly to evaluate consistency with the univariate analyses. In the main text, we present the univariate analysis results and emphasize findings that were supported by both sets of analyses. We provide the model selection results and discuss those findings as supplementary material (Table S4).

### 2.7 Phylogenetic signal

To directly evaluate the extent to which phylogenetic relationships explain expression of these vocal parameters, we computed a phylogenetic signal for each of the component variables that went into the song‐level acoustic space. We calculated Blomberg's *K* using the phylosig function in the phytools R package (Blomberg et al., 2003; Revell, 2012).

## 3 RESULTS

### 3.1 Sex and species differences

**FIGURE 1:**
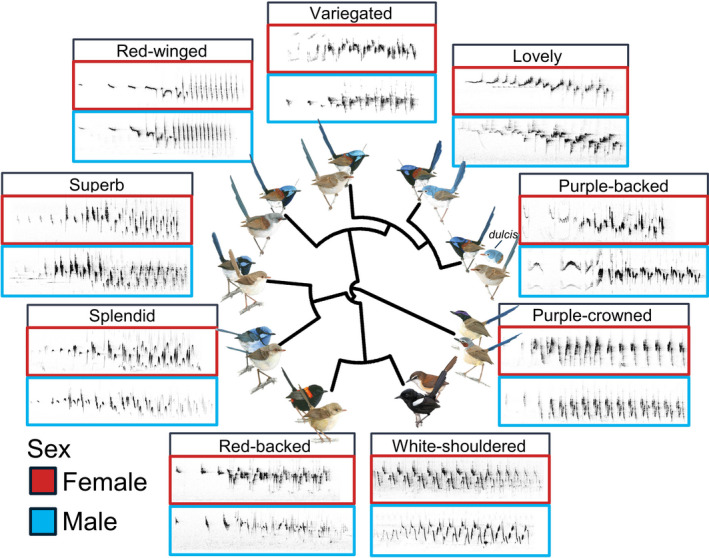
Spectrograms showing male and female songs for nine species of fairy‐wrens. Overall, male and female songs are similar to each other, while overall song structure differs among the nine species. Artwork by Allison E. Johnson

**FIGURE 2:**
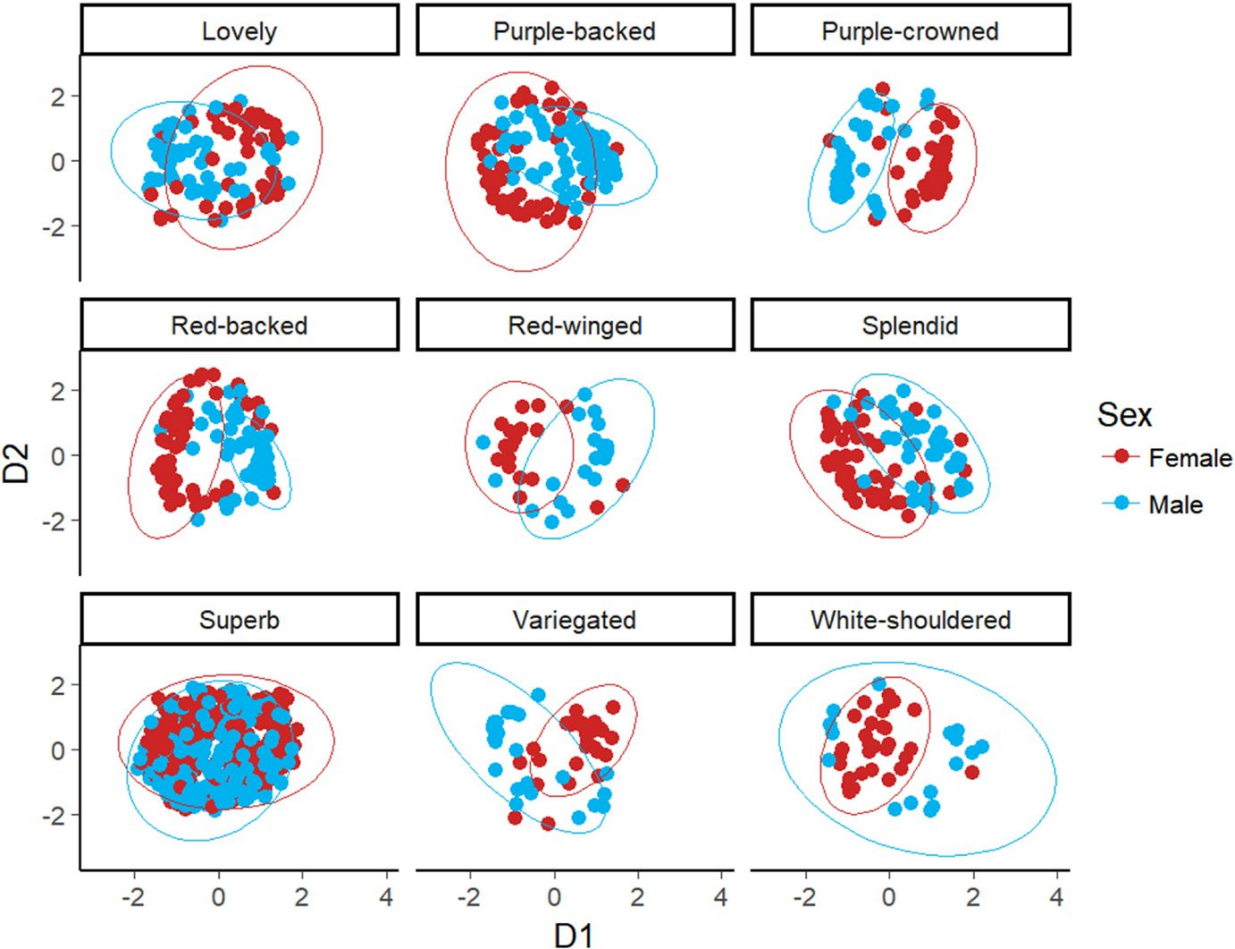
Overlap of male and female songs in acoustic space is similar overall for nine species of fairy‐wren. Male and female songs of certain species (e.g., purple‐crowned, red‐backed, variegated) show some dimorphism based on separation in acoustic space, however, in other species (e.g., lovely, superb) male and female songs are nearly identical. Axes represent the first and second multidimensional scaling (MDS) vectors (D1 and D2)

Male and female fairy‐wren songs were structurally similar within each species, occupying similar regions in acoustic space (Figures 1 and 2). The capacity to reliably classify songs to the correct sex ranged from low in some fairy‐wren species (e.g., superb 61.5%, splendid 62.2%, and red‐winged and variegated 62.5%) to moderately high in others (e.g., purple‐backed 73.4%, purple‐crowned 78.7%, and red‐backed 83.1%; Table 1). In some species, such as white‐shouldered fairy‐wrens, an overall classification of songs to the correct sex was considerably higher for one sex than for the other (90% correct classification for females vs. 25% for males; Table 1), suggesting that songs for one sex encompassed a larger acoustic space which included the acoustic space of the other sex (Figure 2). Overall, fairy‐wren songs could be readily classified to their respective species with a moderately high correct classification of 73.19% (Figures S2 and S3).

**TABLE 1:** Correct classification of fairy‐wren songs to sex, based on random forest classification, including total percent correct for both sexes together and the percent correct for each sex

Species	Total % correct	Female % correct	Male % correct
Superb	61.5%	68.2%	53.5%
Splendid	62.2%	66.0%	57.5%
Red‐winged	62.5%	57.9%	66.7%
Variegated	62.5%	66.7%	57.7%
Lovely	63.3%	64.4%	62.2%
White‐shouldered	67.4%	90.0%	25.0%
Purple‐backed	73.4%	67.3%	77.8%
Purple‐crowned	78.7%	74.3%	82.5%
Red‐backed	83.1%	82.1%	84.1%

The variables that contributed to male–female differences in song varied by species (supplementary materials: Figure S4; Tables S5 and S6). For example, the contribution of each variable to correct classification, as assessed by random forest variable importance rankings indicated that high frequency and element rate contributed most to male and female differences in purple‐crowned fairy‐wrens, whereas song duration, high frequency, and element number contributed most to male–female differences in red‐backed fairy‐wrens (Figure S4; Table S5). More specifically, phylogenetically controlled mixed models of sex differences by species indicated that different variables contributed significantly to male and female differences in song in each fairy‐wren species (Figure S4, Tables S4 and S5). The direction and magnitude of differences between the sexes varied across species (Tables S4 and S5). For example, male red‐backed fairy‐wrens had significantly higher element diversity than females, whereas male white‐shouldered fairy‐wrens had a significantly lower frequency range than females. The acoustic variables contributing to species‐ and sex‐specific differences in song structure were similar across populations within a species; although, some population‐specific variation also existed (random effect estimate of population nested within species = 0.019–0.643 depending on the acoustic variable; Table S5), but note that this effect is confounded with interspecific variation, as we do not have data on replicate populations for every species.

### 3.2 Song elaboration

**FIGURE 3:**
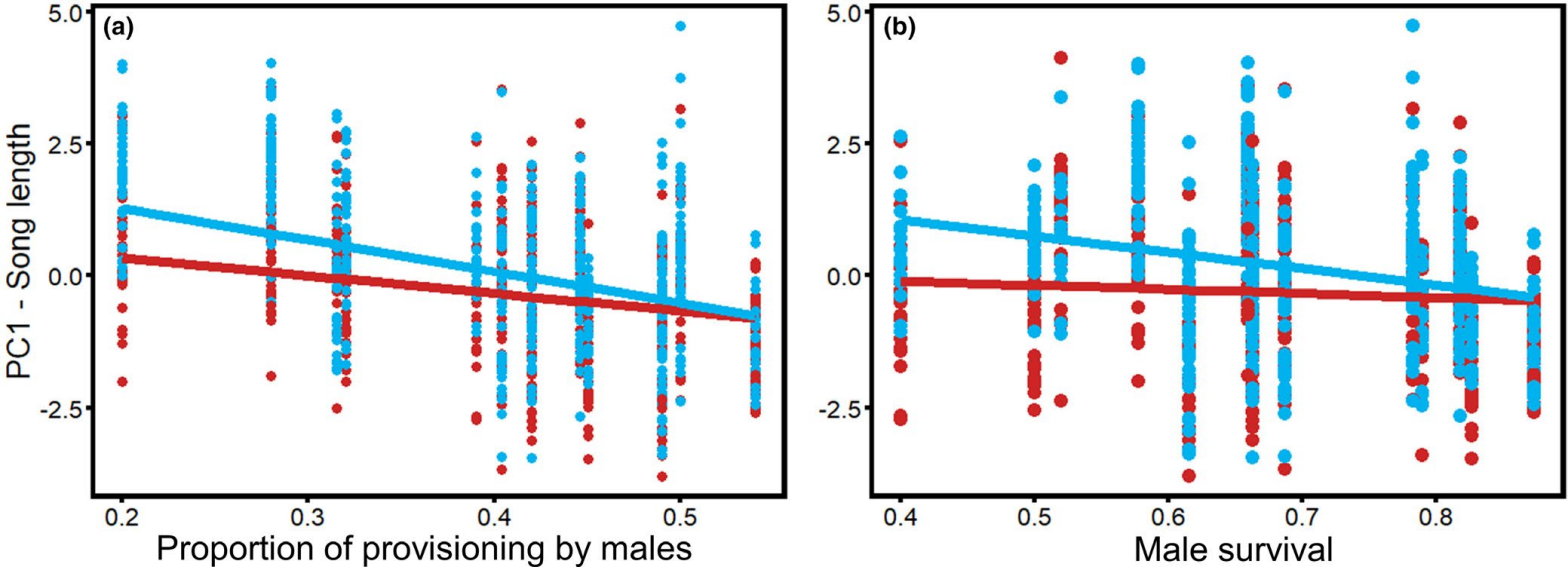
Song length (PC1) compared to (a) proportion of provisioning by the dominant male (compared to the dominant female) and (b) male survival and across 15 fairy‐wren populations. Male songs (blue) were significantly longer than female songs (red) in populations with low male provisioning rates and survival, whereas male and female songs were shorter and similar lengths in populations in which males are long‐lived and provide more equal provisioning compared to females. Each point represents a single song. Trendlines are based on univariate model output

**FIGURE 4:**
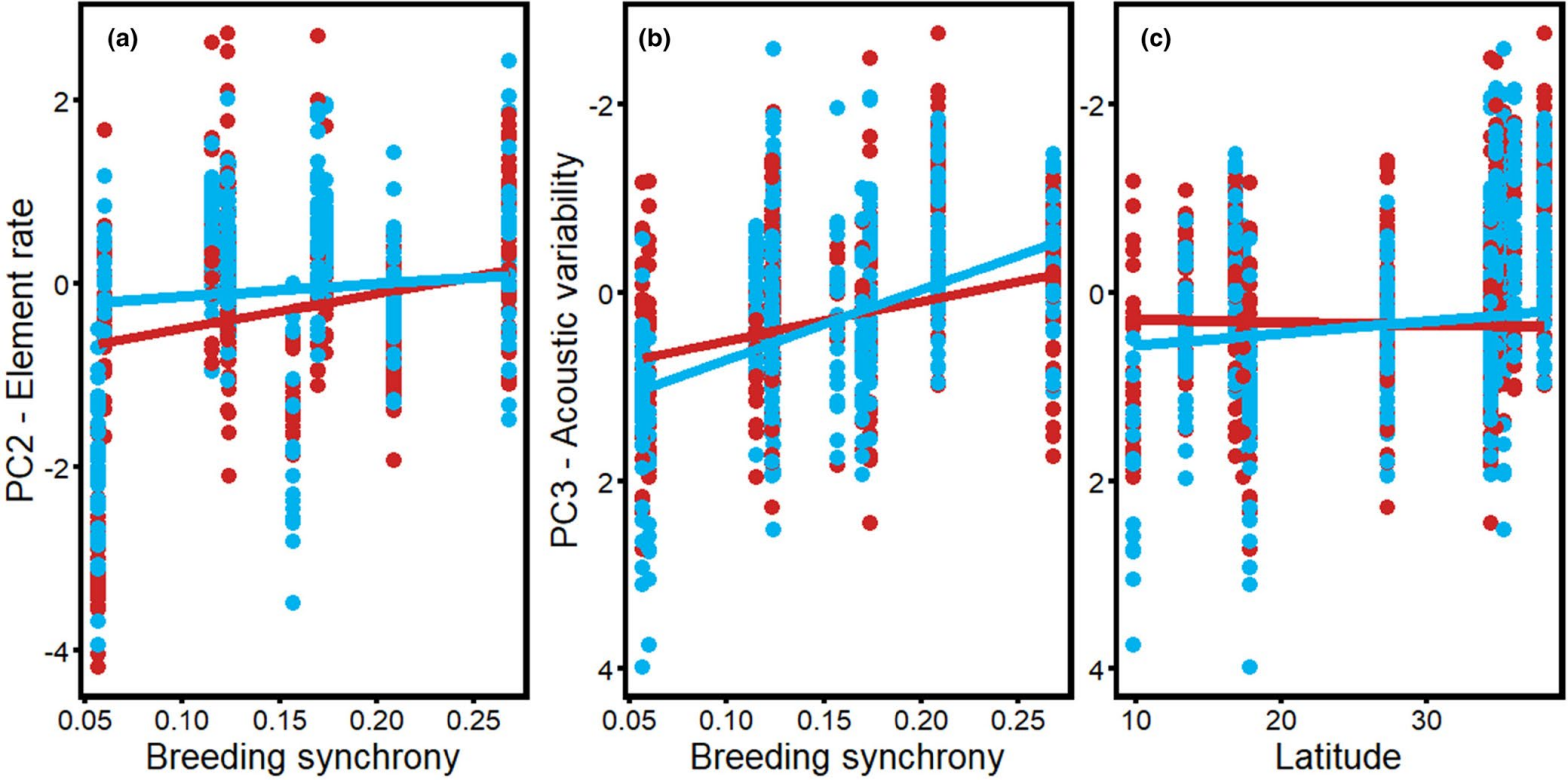
Element rate (PC2) compared to (a) breeding synchrony and song variability (PC3) compared to (b) breeding synchrony and (c) latitude. For PC2, female songs (red) were slower paced than male songs (blue) in less synchronously breeding populations, but similarly paced to male songs in more synchronously breeding populations. For PC3, male song variability (blue) was more positively correlated than female song (red) to these life‐history traits (Table 3). Note that element diversity and frequency range load negatively on PC3 such that more negative values represent more variable songs (Table 2). We flipped the axes on graphs of PC3 so that the relationships with life‐history traits and increasing elaboration can be more readily visualized. Each point represents a single song. Trendlines are based on univariate model output

We quantitated song elaboration via PCA in three components: song length (PC1), element rate (PC2), and song variability (PC3; Table 2 shows loadings for all contributing acoustic variables). Relationships between song elaboration and life‐history traits were sex specific in multiple cases, suggesting that male and female songs have evolved differently in response to certain life‐history traits (Figures 3 and 4; Table 3 and Table S6). For example, male song length (PC1) had a stronger negative association with male survival and male provisioning rates than did female song length (sex * male provisioning: *p* = .009; sex * male survival: *p* = .003). The association between male provisioning rate and song length (PC1) was not recovered by the model selection results (Table S4) likely due to the correlation between male survival and provisioning (*r* = .7; Table S3). Overall, female song length was more consistent across life‐history variables than male song length (Figure 3; Table 3). Specifically, on average, male songs were longer than female songs in populations with low male survival and less male provisioning, whereas male and female songs were of similar lengths and shorter overall in populations in which males are long‐lived and in populations in which males provision offspring at relatively higher rates (Figure 3). There was also a non‐significant interaction between song length and sex, in which males tended to have longer songs than females at tropical latitudes, but shorter, similar length songs at temperate latitudes (*p* = .063; Table 3). In general, fairy‐wrens tended to sing longer songs (PC1) at low latitudes (*p* = .075).

**TABLE 2:** Principal component analysis (A) eigenvalues and the proportion and cumulative variance of each component, and (B) component loadings for each acoustic variable

	PC1—Song length	PC2—Element rate	PC3—Song variability
(A) Eigenvalues and variance			
Standard deviation	1.4385	1.1441	0.9654
Proportion of variance	0.4139	0.2618	0.1864
Cumulative proportion	0.4139	0.6757	0.8620
(B) Loadings			
Duration	**0.538**	−0.416	0.415
Element number	**0.623**	0.202	0.352
Element rate	0.133	**0.823**	‐
Frequency range	0.344	−0.270	**−0.689**
Element diversity	0.432	0.188	**−0.478**

Note

Bold values indicate loadings for variables that contribute >0.5 to each PC.

**TABLE 3:** Phylogenetic mixed model results for song complexity compared to life‐history traits across nine fairy‐wren species, highlighting model results that were significant for life‐history parameters according to Bayesian *p*‐values (***p < .001, **p < .01, *p < .05, *p* < .1)

Response variable	Predictor variable	Posterior mean	Lower 95% CI	Upper 95% CI	Effective sample size	Bayesian *p*‐value
PC1 (Song length)	Male feeding rates	−3.39	−8.29	1.80	4754	.189
	Sex: male	1.47	0.68	2.29	9990	<.001***
	Male feeding rates—Sex: male	−2.65	−4.58	−0.64	9990	.009**
	Male survival	−0.77	−4.51	2.85	6109	.681
	Sex: male	2.10	1.09	3.16	9456	<.001***
	Male survival—Sex: male	−2.34	−3.86	−0.87	9584	.003**
	Latitude	−0.03	−0.07	0.00	9990	.075.
	Sex: male	1.00	0.42	1.61	9990	.001***
	Latitude—Sex: male	−0.02	−0.04	0.00	9281	.063.
PC2 (Element rate)	Breeding synchrony	3.43	−7.78	16.45	1022	.684
	Sex: male	0.60	0.26	0.94	9990	.001***
	Breeding synchrony—Sex: male	−2.46	−4.54	−0.28	9990	.024*
	Latitude	−0.05	−0.11	0.00	2098	.076.
	Sex: male	0.16	−0.17	0.51	9990	.353
	Latitude—Sex: male	0.00	−0.01	0.01	9990	.837
PC3 (Song variability)	Breeding synchrony	−4.25	−9.34	0.56	7909	.072.
	Sex: male	0.51	0.15	0.91	9990	.010*
	Breeding synchrony—Sex: male	−3.17	−5.60	−0.90	9990	.008*
	Latitude	0.00	−0.03	0.03	4836	.720
	Sex: male	0.43	0.04	0.81	9990	.027*
	Latitude—Sex: male	−0.02	−0.03	0.00	9990	.019*

Note

See Table S6 for a full set of model results. Values in bold are results that are supported by both univariate model and best model results. Note that PC3 loadings are negative, so more negative values for PC3 reflect more variable songs.

Element rate (PC2) and song variability (PC3) also exhibited sex‐specific patterns, with male songs showing stronger, positive relationships to variables tied to mate competition, whereas female songs were more consistent across variation in life‐history traits (Table 3). Specifically, element rate (PC2) exhibited an interaction between sex and breeding synchrony such that female songs were slower paced than male songs in less synchronously breeding populations, whereas male and female element rates were similar in more synchronously breeding populations (sex * breeding synchrony *p* = .024; Figure 4a). As for song variability (PC3), male song variability had a stronger positive association than female song variability with breeding synchrony (*p* = .080) and latitude (*p* = .019). Interestingly, female songs were more variable than male songs in populations with high breeding synchrony and at high latitudes (*p *< .027, Table 3). Conversely, male songs were more variable than female songs in more synchronously breeding populations and songs of both sexes were more similarly variable at higher latitudes (Figure 4b,c). Both males and females tended to sing faster paced songs (PC2) at low latitudes (*p* = .076), and their songs tended to be more variable (PC3) in more synchronously breeding populations (*p* = .072), especially in males (*sex, *p* = .008; Figure 3; Table 3).

### 3.3 Song dimorphism

**FIGURE 5:**
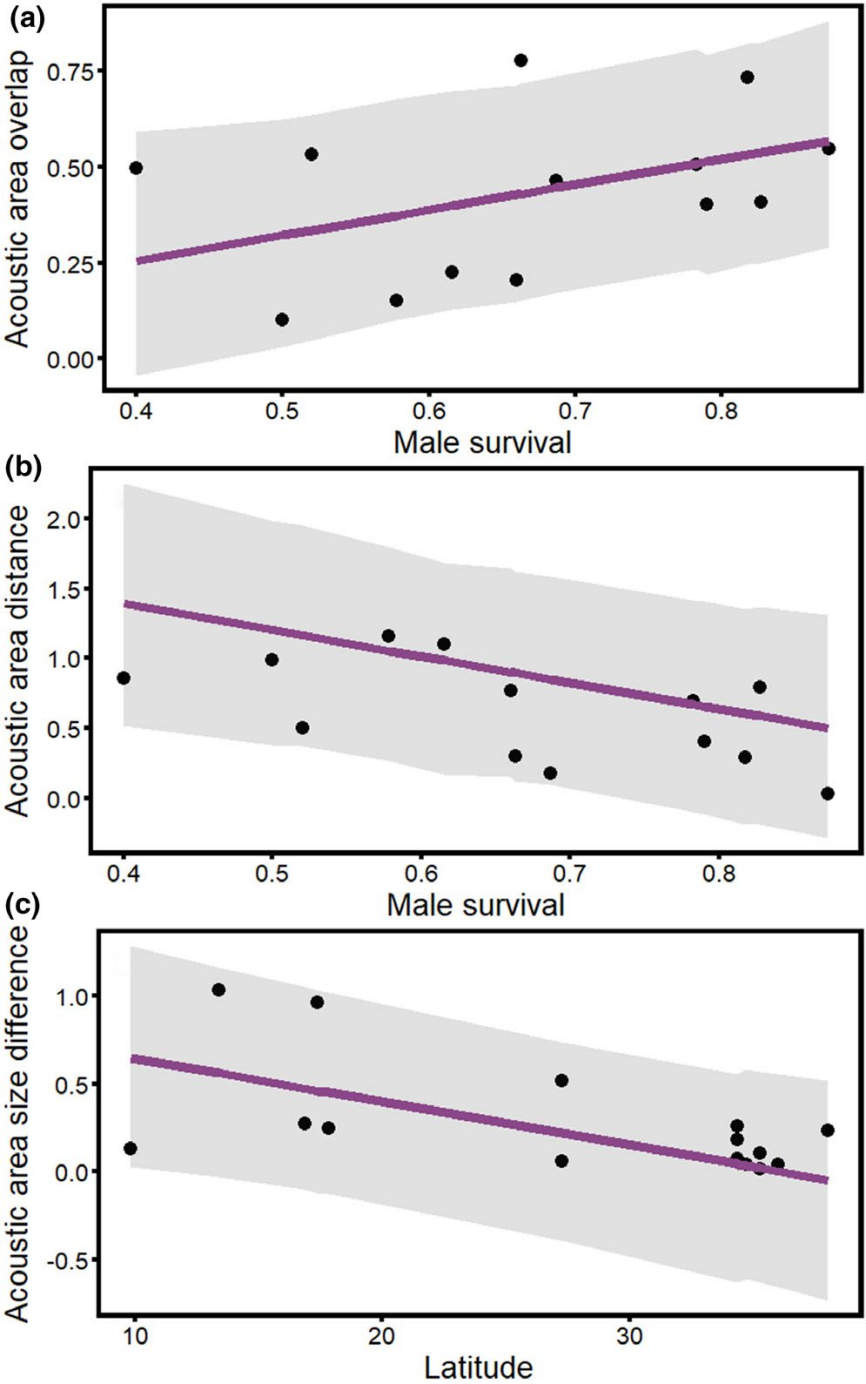
Sexual song dimorphism compared to life‐history traits for 15 populations of fairy‐wrens. Male and female (a) acoustic areas overlapped most and (b) were closest together in populations with high male survival. Male and female (c) acoustic areas were similarly variable at high latitudes. Each point represents song dimorphism for a single species. Trendlines are based on univariate model output

Male and female song similarity was correlated with variables linked to sex role convergence, consistent with the differences between the sexes in elaboration found above. Male and female songs were most similar, measured as both overlap and distance in acoustic space, in populations with high male survival (*p* = .022 & *p* = .025, respectively; Figure 5; Table 4). There was also a non‐significant trend for male and female songs to be more similar when the proportion of provisioning by males was higher, (i.e., when male and female provisioning was more equal; *p* = .082). Interestingly, fairy‐wrens also exhibited patterns contrary to any hypotheses that we considered: male and female songs were similarly variable (had similarly sized acoustic areas) at temperate latitudes, whereas sexual dimorphism in song variability was higher at more tropical latitudes (*p *= .036; Figure 5; Table 4), consistent with the song elaboration findings above (c.f. Figure 4c). In addition, model selection results recovered positive correlations between male–female overlap in acoustic space and extra‐pair paternity (EPP) rates (*p* = .030; higher EPP rates in species with more monomorphic songs) and male–female differences in song variability and breeding synchrony (*p* = .039; marginally higher breeding synchrony in species with similar male–female song variability; Table S4).

**TABLE 4:** Phylogenetic mixed model results for song dimorphism compared to life‐history traits across nine fairy‐wren species

Response variable	Predictor variable	Posterior mean	Lower 95% CI	Upper 95% CI	Effective sample size	Bayesian *p*‐value
Male–female overlap in acoustic area	Percent extra‐pair paternity	0.39	−0.12	0.87	16,650	.093.
	Brood size	−0.14	−0.37	0.06	16,650	.167
	Group size	0.00	−0.17	0.17	16,650	1.000
	Provisioning by male	0.82	−0.54	2.18	16,650	.210
	Male survival	0.65	0.16	1.19	16,154	.022*
	Breeding synchrony	0.02	−1.88	2.17	16,650	.954
	Latitude	0.00	−0.01	0.01	17,718	.801
	Breeding male density	2.75	−4.43	10.21	16,650	.420
Male–female distance in acoustic area	Percent extra‐pair paternity	−0.79	−2.84	1.23	17,685	.403
	Brood size	0.29	−0.45	1.03	17,695	.410
	Group size	−0.04	−0.55	0.45	14,849	.890
	Provisioning by male	−2.79	−6.03	0.40	16,650	.082.
	Male survival	−1.89	−3.52	−0.29	16,650	.025*
	Breeding synchrony	−1.27	−7.90	3.68	11,874	.820
	Latitude	−0.01	−0.04	0.03	16,650	.725
	Breeding male density	−9.09	−27.94	8.83	16,650	.271
Male–female acoustic area size difference	Percent extra‐pair paternity	−1.09	−2.70	0.48	16,049	.157
	Brood size	0.03	−0.50	0.55	17,832	.920
	Group size	−0.12	−0.40	0.17	17,312	.350
	Provisioning by male	0.41	−1.88	2.59	15,907	.710
	Male survival	−0.50	−2.06	0.98	15,966	.490
	Breeding synchrony	0.78	−2.96	4.49	16,650	.650
	Latitude	−0.03	−0.05	0.00	15,528	.036*
	Breeding male density	−1.28	−15.97	12.47	16,870	.850

Note

***p ≤ .001, **p ≤ .01, *p ≤ .05, *p* ≤ .1 according to Bayesian *p*‐values. Values in bold are results that are supported by both univariate model and best model results (See Table S4 for more details).

### 3.4 Phylogenetic signal

Song duration and element number had a high phylogenetic signal (*K* = 1.35 and 1.42, respectively), indicating that these song features are most similar among closely related species and could be constrained by shared ancestry. Element diversity (*K* = 0.80), frequency range (*K* = 0.87), and element rate (*K* = 0.91) had an intermediate phylogenetic signal, indicating that these traits are more similar in closely related species and phylogeny could play some role in shaping these traits, but that they also evolve somewhat independently of phylogeny. For the high‐ and low‐frequency variables, however, the phylogenetic signal was lower than expected under Brownian motion (*K* appreciably less than one: frequency 5%, *K* = 0.69; frequency 95%, *K* = 0.58), suggesting that these aspects of song have not been constrained by phylogenetic relationships.

## 4 DISCUSSION

Male and female fairy‐wren songs were generally structurally similar, but sexual song dimorphism was greater in some fairy‐wren species than in others. The song parameters that differed between males and females varied widely across species. Song elaboration was weakly related to life‐history variables overall, but showed some sex‐specific relationships, suggesting that different selective pressures have shaped some aspects of song elaboration in each sex. Trends were consistent with stronger sexual selection on male songs than on female songs in some populations and species. In contrast, sexual song dimorphism was associated with life‐history traits suggesting an association between song dimorphism and divergence in sex roles: in long‐lived species, which also had higher paternal care, males and females also had more similar songs. Finally, some aspects of song structure showed a strong phylogenetic signal, whereas others differed even between closely related species, suggesting there could be evolutionary constraints on female and male fairy‐wren songs. We conclude that song elaboration and sexual song dimorphism across fairy‐wrens has been shaped by diverse selective pressures, including sexual selection and selection favoring sex role similarity. Lastly, we did not detect a positive correlation between song complexity and latitude, as has been observed in the Northern Hemisphere. Thus, in Southern Hemisphere species, the association between latitude, life‐history traits, and tropical–temperate gradients may be weaker or absent.

### 4.1 Song elaboration

Our results suggest that song elaboration in fairy‐wrens has been shaped at least in part by sexual selection acting more strongly on the male song than on the female song in certain situations. Male song variability increased more strongly with breeding synchrony and latitude than did female song variability (Figure 4b,c). Interestingly, females had slower paced songs than males in populations with low breeding synchrony (Figure 4c) and shorter songs than males in short‐lived species and when males provisioned less (Figure 3). However, female songs were more variable than male songs in populations with low breeding synchrony (Figure 4b). The stronger correlation of male song elaboration with factors germane to mating opportunities supports the idea that song elaboration has been sexually selected in males, but not females. This finding is consistent with Greig et al.’s (2013) finding that several aspects of male fairy‐wren type 1 song were correlated with testes mass. Nevertheless, we did not find any relationship between male or female song elaboration and extra‐pair paternity rates, as a potential predictor of sexual selection. This is consistent with meta‐analyses and other comparative studies of song which also did not find correlations between song elaboration and extra‐pair paternity rates (Garamszegi & Møller, 2004; Soma & Garamszegi, 2011). To our knowledge no studies have looked for similar patterns in female song. Field studies should follow up on our findings to determine whether these are aspects of male and/or female songs that fairy‐wrens attend to, and whether variation in these traits is associated with differential mating success in males or reproductive success in females. In addition, such studies should gather metrics of reproductive success that are applicable to females (e.g., fecundity or fledgling success), as EPP is probably not as meaningful a metric of reproductive success in females as in males.

Our principal component results combined with our dimorphism results suggest that song elaboration in fairy‐wrens may also be associated with selective pressures that have favored sex role similarity or constraints on female song length. For example, song length was more highly correlated with lower provisioning and reduced survival in males than in females. However, male and female songs were both shorter and more similar overall when male and female provisioning was more equal and male survival was high (Figure 3). These patterns are consistent with divergent selective pressures or different strengths of selective pressures having resulted in shorter, monomorphic songs in some populations and longer, dimorphic songs in other populations (Catchpole, 1982; Najar & Benedict, 2019). Conversely, natural selection against elaborate female songs could have constrained female song length in populations where females provide most of the provisioning. Nest predation has been tied to high song rates in the nest in female superb fairy‐wrens (Kleindorfer et al., 2016) and would be consistent with the patterns we found (but see Cain et al., 2015; Cain & Langmore, 2016). Moreover, female songs have similar variability among species than male songs, and female songs are actually more variable than male songs in populations with lower breeding synchrony (Figure 4). This suggests that there could have been a selection to maintain high variability of female songs within fairy‐wrens. In superb fairy‐wrens, the songs of young males and females share similar element diversity with their parents (Evans & Kleindorfer, 2016). If element diversity helps dispersing females establish a territory, then it is possible that only females with more diverse element repertoires are able to acquire territories, consistent with the pattern we observe. Past research has focused largely on directional selection toward male song diversity and elaboration. Recent research suggests, however, that bird song is likely under balancing selection for intermediate‐sized song repertoires (Snyder & Creanza, 2019). Our results are consistent with multiple selective pressures acting on the songs of both sexes, resulting in particularly elaborate male songs in certain conditions, but intermediately elaborated songs in both sexes in others.

Sex differences in song elaboration may also reflect different intensities of sexual selection acting on male and female songs. Specifically, intra‐sexual selection may have been important in both sexes, whereas inter‐sexual selection may have been more important in males. This could also be due in part to correlated evolution of female song structure with male song structure, coupled with relaxation of selection on female song traits (Lande, 1980). However, functional studies in fairy‐wrens suggest that though song is sexually selected in males, it is also important in intra‐sexual competition in females (Cooney & Cockburn, 1995). Female fairy‐wren song is used for territory defense, and the consequences of losing, or failing to gain, a territory may be more dire for females than males (Cain & Langmore, 2015; Colombelli‐Négrel, 2016; Cooney & Cockburn, 1995; Enbody et al., 2018; Leitao, 2019). Moreover, females that sing and exhibit greater aggression to simulated intrusions also have higher reproductive success, although the relationship varies according to habitat quality (Cain et al., 2015; Cain & Langmore, 2016). Therefore, a female song is functional with known reproductive benefits. Song structure in female fairy‐wrens may thus be a balance between the use of song to enhance offspring quality and win competitive interactions versus the costs associated with certain aspects of a female song (Cain & Rosvall, 2014; Kleindorfer et al., 2016).

### 4.2 Sex differences & song dimorphism

Our results also provide strong evidence that fairy‐wren song structure has been shaped by selective pressures favoring sex role similarity. Male and female songs were shorter and more similar when males and females provision more equally and when male survival is high (in longer lived species; Figure 3). Therefore, patterns of song dimorphism are likely not just a by‐product of strong, directional selection on males, but rather a balance between sexual selection, natural selection, and active maintenance of sex role similarity in certain populations. This may represent two sides of the same coin—strong directional selection for particularly elaborate male traits in polygynous species versus reduced trait elaboration in monogamous species with male care are extremes on a sexual selection continuum (Bradbury et al., 2000). Plus, traits in both sexes can be sexually selected through mutual mate choice (Johnstone et al., 1996). However, to understand selective pressures responsible for sexual dimorphism (as opposed simply to trait elaboration), we need to independently investigate selective pressures that act on both males and females (Price, 2015). This is particularly true if we want to elucidate the selective pressures responsible for sexual dimorphism from a mutually ornamented ancestor (Odom et al., 2014). Our findings provide evidence that selection has favored both dimorphism and sexual monomorphism in song under different circumstances. Specifically, male and female songs are similar in long‐lived species and female songs are faster paced in more synchronously breeding species, scenarios that could lead to increased intra‐sexual competition for territories or non‐mate breeding resources, which could be conceived as social selection (e.g., Tobias et al., 2012).

Sex‐specific songs may also be selected for by factors other than those related to sexual selection and sex role similarity. Interestingly, three species known to regularly duet, the purple‐crowned, red‐backed, and white‐shouldered fairy‐wrens (Rowley & Russell, 1997), also had some of the most sexually dimorphic songs. This is consistent with the pattern in some other major lineages of duetting species, which often have sex‐specific songs (e.g., Cisticolidae: Thorpe et al., 1972; Malaconotidae: Grafe & Bitz, 2004; Van Den Heuvel et al., 2013; Troglodytidae: Logue et al., 2007; Mann et al., 2009). This suggests that duetting species may have experienced additional selective pressures for sex‐specific song structure. In the Neotropical wrens (Troglodytes), a family well‐known for their highly coordinated duets, distinct male and female song structure, has been hypothesized to allow rapid sex or identity signaling when both sexes defend territories cooperatively (Hall, 2004; Logue et al., 2007). Therefore, sexual selection and sex role similarities might not be the only selective pressures that have resulted in the dimorphic songs observed between female and male fairy‐wrens. Frameworks to explain sexual dimorphism in song structure should take these additional functions and selective pressures of sex‐specific song into account, as they may be important in cases where tropical or sedentary species display sexually dimorphic signals (for example, Grafe & Bitz, 2004; Logue et al., 2007; Mays et al., 2006; Rivera‐Cáceres et al., 2018).

The acoustic parameters driving male and female fairy‐wren song dimorphism differed across species. Based on random forest variable importance scores, frequency parameters (low frequency, high frequency, or frequency bandwidth) contributed to classification of males and females in most species, which is not unexpected given the sexes typically differ slightly in body size. However, size dimorphism in fairy‐wrens is minimal (Rowley & Russell, 1997). Furthermore, element diversity, song duration, and element rate also contributed considerably to sex differences (Figure S4). Therefore, the features of songs that capture male–female dimorphism appear to be very species specific, possibly as a result of character displacement or the role different features play in mate recognition (Grant, 1972; Price, 2007; Queller & Strassmann, 2018). As a group, fairy‐wren songs are fairly similar across species, so the fact that different variables contribute to sex differences in this clade suggests that the features that explain song dimorphism are probably quite variable across all species, especially in more distantly related taxa. Therefore, it may be important to measure a wide range of features when trying to assess sexual dimorphism in song (Benedict & Najar, 2019).

### 4.3 Latitude, song, and the Southern Hemisphere

Hypotheses based on Northern Hemisphere species predict that song elaboration will be greatest in higher latitudes (Catchpole, 1982; Najar & Benedict, 2019; Read & Weary, 1992). However, instead we found that male songs tended to be shorter and male and female songs were more similar and less variable at higher (temperate) latitudes. This pattern adds to other recent findings that predicted relationships between latitude and complexity are not always empirically supported, particularly when applying those based on Northern Hemisphere species to southern species (Najar & Benedict, 2019). Nevertheless, we did find support for the hypothesis that both selection for similar sex roles and sexual selection pressures have shaped fairy‐wren song structure. Therefore, we found support for the evolutionary mechanisms for which latitude is expected to be a proxy (trade‐offs in sexual vs. natural selection). We suggest that this is because the relationship between song complexity and latitude may reflect Northern Hemisphere life‐history patterns, which may not apply or be as pronounced in the Southern Hemisphere. Many northern temperate songbirds are migratory (latitudinal or altitudinal) and are only seasonally territorial. In contrast to north temperate climate patterns, winter temperatures in Australia are often more moderate and many species, including fairy‐wrens, remain on their territories year‐round (Del Hoyo et al., 2010; Rowley & Russell, 1997). In addition, fairy‐wrens are socially monogamous, cooperative breeders with considerable variation in their reproductive strategies, a combination that is uncommon in north temperate regions (Cockburn, 2003; Feeney et al., 2013; Jetz & Rubenstein, 2011). Therefore, for a variety of reasons, Southern Hemisphere species, and specifically fairy‐wrens, may contradict expected latitudinal life‐history patterns associated with north temperate latitudes (see Table S2 for low correlations between latitude and survival, male provisioning rates, and breeding synchrony). For these reasons, our study system offers a unique opportunity to tease apart underlying selective pressures dictating patterns of bird song elaboration and dimorphism, irrespective of climate and latitude. We expect that our findings broadly reflect the underlying balance of selective pressures expected to shape male and female bird song evolution generally.

## 5 CONCLUSIONS

Understanding the selective forces that have shaped trait elaboration and sex differences across species is a key issue for evolutionary biologists and requires detailed data on life histories and trait variation. Drawing on such data, we provide evidence that similar forces have shaped female and male song elaboration in a well‐studied Southern Hemisphere clade, and that these forces are to some extent decoupled from the latitudinal patterns predicted from Northern Hemisphere studies. Furthermore, we find evidence for sex differences in the elaboration of song that are consistent with sexual selection theory, with males in some instances having more elaborate songs than females. Yet our results are also consistent with social selection for consistently elaborate songs in females and possibly natural selection for shorter female songs in some species. Moreover, selective pressures that favor more similar sex roles also appear to have shaped song structure in both sexes, providing evidence that complex songs in fairy‐wrens are not driven primarily by inter‐sexual selection. Instead, our findings indicate that song structure may be an interplay of both sexual selection on males and selective pressures that mediate sex role similarity, and that song dimorphism results from the balance between these two evolutionary forces.

## CONFLICT OF INTEREST

This study was supported by funding sources cited in the Acknowledgments. The authors have no conflicts of interest.

## AUTHOR CONTRIBUTIONS

**Karan J. Odom:** Conceptualization (lead); data curation (lead); formal analysis (lead); funding acquisition (lead); investigation (lead); methodology (lead); project administration (lead); resources (lead); software (lead); supervision (lead); validation (lead); visualization (lead); writing‐original draft (lead); writing‐review & editing (lead). **Kristal E. Cain:** Conceptualization (equal); data curation (equal); formal analysis (equal); funding acquisition (equal); investigation (equal); methodology (equal); project administration (equal); resources (equal); validation (equal); visualization (equal); writing‐original draft (equal); writing‐review & editing (equal). **Michelle L. Hall:** Conceptualization (equal); data curation (equal); formal analysis (equal); funding acquisition (equal); investigation (equal); methodology (equal); project administration (equal); resources (equal); supervision (equal); validation (equal); visualization (equal); writing‐original draft (equal); writing‐review & editing (equal). **Naomi E. Langmore:** Conceptualization (equal); data curation (equal); formal analysis (equal); funding acquisition (equal); investigation (equal); methodology (equal); project administration (equal); resources (equal); supervision (equal); validation (equal); visualization (equal); writing‐original draft (equal); writing‐review & editing (equal). **Raoul A. Mulder:** Conceptualization (supporting); data curation (supporting); formal analysis (supporting); funding acquisition (equal); investigation (supporting); methodology (supporting); project administration (supporting); resources (equal); supervision (supporting); validation (supporting); visualization (supporting); writing‐original draft (supporting); writing‐review & editing (supporting). **Sonia Kleindorfer:** Conceptualization (supporting); data curation (supporting); formal analysis (supporting); funding acquisition (supporting); investigation (supporting); methodology (supporting); project administration (supporting); resources (supporting); supervision (supporting); validation (supporting); visualization (supporting); writing‐original draft (supporting); writing‐review & editing (supporting). **Jordan Karubian:** Conceptualization (supporting); data curation (supporting); formal analysis (supporting); funding acquisition (supporting); investigation (supporting); methodology (supporting); project administration (supporting); resources (supporting); supervision (supporting); validation (supporting); visualization (supporting); writing‐original draft (supporting); writing‐review & editing (supporting). **Lyanne Brouwer:** Conceptualization (supporting); data curation (equal); formal analysis (supporting); funding acquisition (supporting); investigation (supporting); methodology (supporting); project administration (supporting); resources (supporting); supervision (supporting); validation (equal); visualization (supporting); writing‐original draft (supporting); writing‐review & editing (supporting). **Erik D. Enbody:** Conceptualization (supporting); data curation (supporting); formal analysis (supporting); funding acquisition (supporting); investigation (supporting); methodology (supporting); project administration (supporting); resources (supporting); supervision (supporting); validation (supporting); visualization (supporting); writing‐original draft (supporting); writing‐review & editing (supporting). **John Anthony Jones:** Conceptualization (supporting); data curation (supporting); formal analysis (supporting); funding acquisition (supporting); investigation (supporting); methodology (supporting); project administration (supporting); resources (supporting); supervision (supporting); validation (supporting); visualization (supporting); writing‐original draft (supporting); writing‐review & editing (supporting). **Jenélle L. Dowling:** Conceptualization (supporting); data curation (supporting); formal analysis (supporting); funding acquisition (supporting); investigation (supporting); methodology (supporting); project administration (supporting); resources (supporting); supervision (supporting); validation (supporting); visualization (supporting); writing‐original draft (supporting); writing‐review & editing (supporting). **Ana V. Leitão:** Conceptualization (supporting); data curation (supporting); formal analysis (supporting); funding acquisition (supporting); investigation (supporting); methodology (supporting); project administration (supporting); resources (supporting); supervision (supporting); validation (supporting); visualization (supporting); writing‐original draft (supporting); writing‐review & editing (supporting). **Emma I Greig:** Conceptualization (supporting); data curation (supporting); formal analysis (supporting); funding acquisition (supporting); investigation (supporting); methodology (supporting); project administration (supporting); resources (supporting); supervision (supporting); validation (supporting); visualization (supporting); writing‐original draft (supporting); writing‐review & editing (supporting). **Christine Evans:** Conceptualization (supporting); data curation (supporting); formal analysis (supporting); funding acquisition (supporting); investigation (supporting); methodology (supporting); project administration (supporting); resources (supporting); supervision (supporting); validation (supporting); visualization (supporting); writing‐original draft (supporting); writing‐review & editing (supporting). **Allison E. Johnson:** Conceptualization (supporting); data curation (supporting); formal analysis (supporting); funding acquisition (supporting); investigation (supporting); methodology (supporting); project administration (supporting); resources (supporting); supervision (supporting); validation (supporting); visualization (supporting); writing‐original draft (supporting); writing‐review & editing (supporting). **Kimberley K.‐A. Meyers:** Data curation (supporting); writing‐original draft (supporting); writing‐review & editing (supporting). **Marcelo Araya‐Salas:** Conceptualization (equal); data curation (equal); formal analysis (lead); funding acquisition (supporting); investigation (equal); methodology (equal); project administration (equal); resources (equal); software (lead); supervision (equal); validation (lead); visualization (lead); writing‐original draft (equal); writing‐review & editing (equal). **Michael S. Webster:** Conceptualization (equal); data curation (equal); formal analysis (equal); funding acquisition (equal); investigation (equal); methodology (equal); project administration (equal); resources (equal); supervision (equal); validation (equal); visualization (equal); writing‐original draft (equal); writing‐review & editing (equal).

## Supporting information

Fig S1‐S5

Table S1‐S6

## Data Availability

Code and data for these analyses are available at dryad.org: https://doi.org/10.5061/dryad.n8pk0p2ws. Select songs measured for the acoustic analysis have been made available at macaulaylibrary.org.
