# Supplementary material for: Sex role similarity and sexual selection predict male and female song elaboration and dimorphism in fairy‐wrens
Source: Ecol Evol. 2021 Dec 7;11(24):17901–19. doi: 10.1002/ece3.8378 (PMC8717346; doi:10.1002/ece3.8378)

**Supplementary figures for:**

**Title:** Sex role similarity and sexual selection predict male and female song elaboration and dimorphism in fairy-wrens

**Journal:** Ecology and Evolution

**Year:** 2021

**Authors:** Karan J. Odom^1, 2^*, Kristal E. Cain^3,4^, Michelle L. Hall^5,6,7, 8^, Naomi E. Langmore^3^, Raoul A. Mulder^5^, Sonia Kleindorfer^9.10^, Jordan Karubian^11^, Lyanne Brouwer^3,12,13^, Erik D. Enbody^11,14^, John Anthony Jones^11^, Jenélle L. Dowling^15^, Ana V. Leitão^5^, Emma I. Greig^16^, Christine Evans^9^, Allison E. Johnson^17^, Kimberley K.-A. Meyers^5^, Marcelo Araya-Salas^1,18^ and Michael S. Webster^1^

**Author affiliations:**

1. Cornell Lab of Ornithology and Department of Neurobiology and Behavior, Cornell University, Ithaca, New York 14850 USA
2. Department of Psychology, University of Maryland, College Park, College Park, Maryland 20742, USA
3. Research School of Biology, Australian National University, Canberra, ACT, 0200, Australia
4. School of Biological Sciences, University of Auckland, Auckland NZ
5. School of BioSciences, The University of Melbourne, Melbourne, Victoria, 3010, Australia
6. Bush Heritage Australia, Level 1/395 Collins Street, Melbourne, Victoria, 3000
7. The University of Western Australia, 35 Stirling Highway, Perth, Western Australia, 6009, Australia
8. Max Planck Institute for Ornithology, Vogelwarte Radolfzell, D-78315, Germany
9. College of Science and Engineering, Flinders University, Adelaide, South Australia, 5001
10. Konrad Lorenz Research Center for Behaviour and Cognition, Department of Behavioural and Cognitive Biology, University of Vienna, Vienna, Austria, 1090
11. Tulane University, Department of Ecology and Evolutionary Biology, 6823 St. Charles Ave., 400 Lindy Boggs, New Orleans, LA 70118, USA
12. Department of Animal Ecology & Physiology, Institute for Water and Wetland Research, Radboud University, Nijmegen, the Netherlands
13. Department of Animal Ecology, Netherlands Institute of Ecology (NIOO-KNAW), Wageningen, the Netherlands
14. ﻿Department of Medical Biochemistry and Microbiology, Uppsala University, SE-751 23 Uppsala, Sweden
15. Division of Biological Sciences, University of Montana, Health Sciences 203, Missoula, MT 59812 USA
16. Cornell Lab of Ornithology and Project Feeder Watch, Cornell University, Ithaca, New York 14850 USA
17. School of Biological Sciences, University of Nebraska-Lincoln, Lincoln, Nebraska 68588 USA
18. Sede del Sur, Universidad de Costa Rica, 14, Puntarenas, Golfito, Costa Rica

**Figure S1.** A map of the sampling locations by name, with species indicated by colored circles.

**Figure S2.** A two-dimensional acoustic space illustrating how songs group for the nine fairy-wren species from 15 populations. The feature space was created by plotting the first two multidimensional scaling (MDS) coordinates (D1 and D2).

**Figure S3.** Correct classification for all nine fairy-wren species within the study compared to each other species.

**Figure S4.** Variable importance scores indicating the contribution of each song-level parameter to correct classification of males and females to each species acoustic space. Scores were calculated from supervised random forest analysis conducted separately for each species.

**Figure S5.** Model diagnostics: Gelman–Rubin convergence diagnostic, trace plots, and autocorrelation for best MCMCglmm model results.


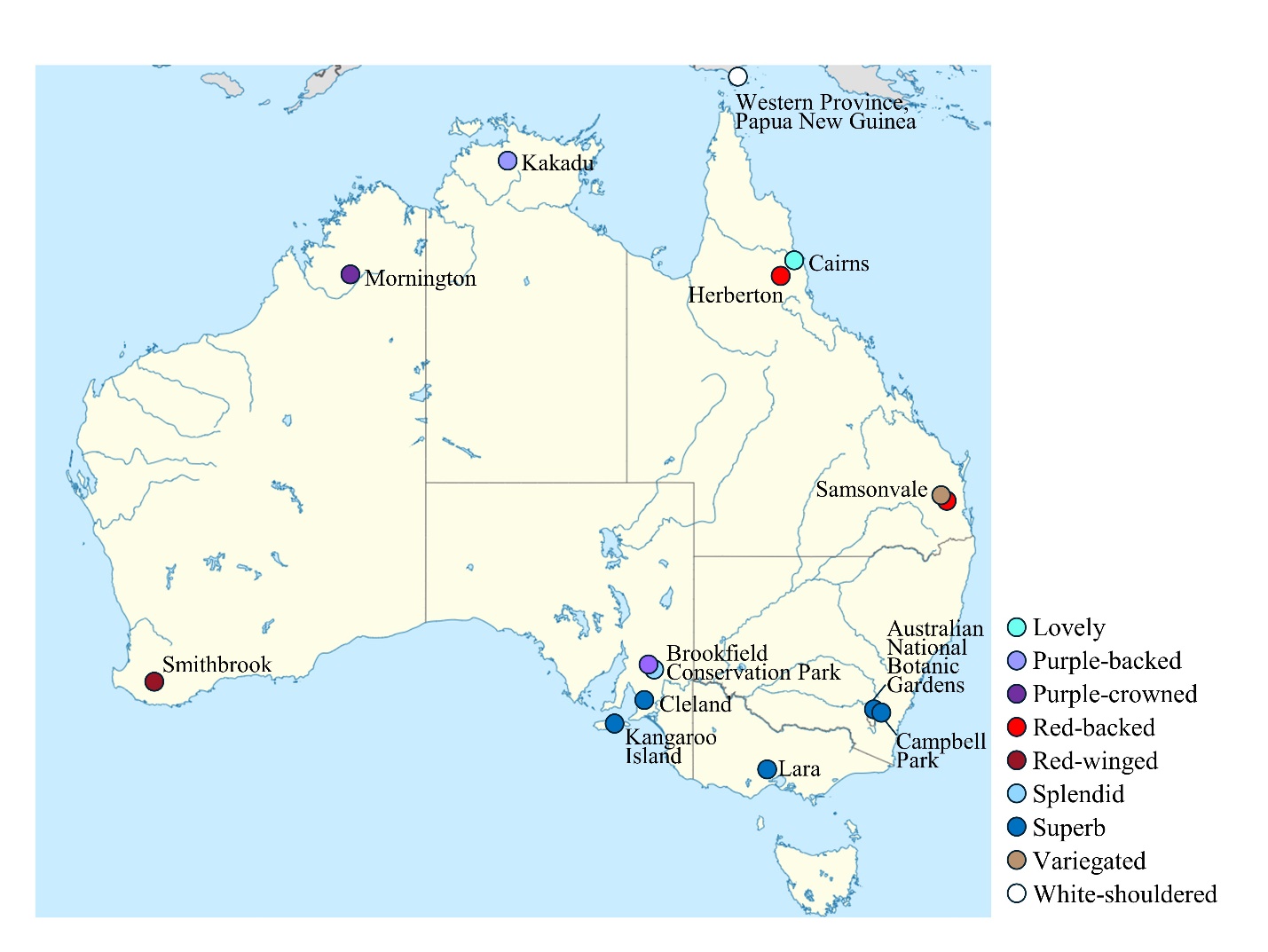


Figure S1


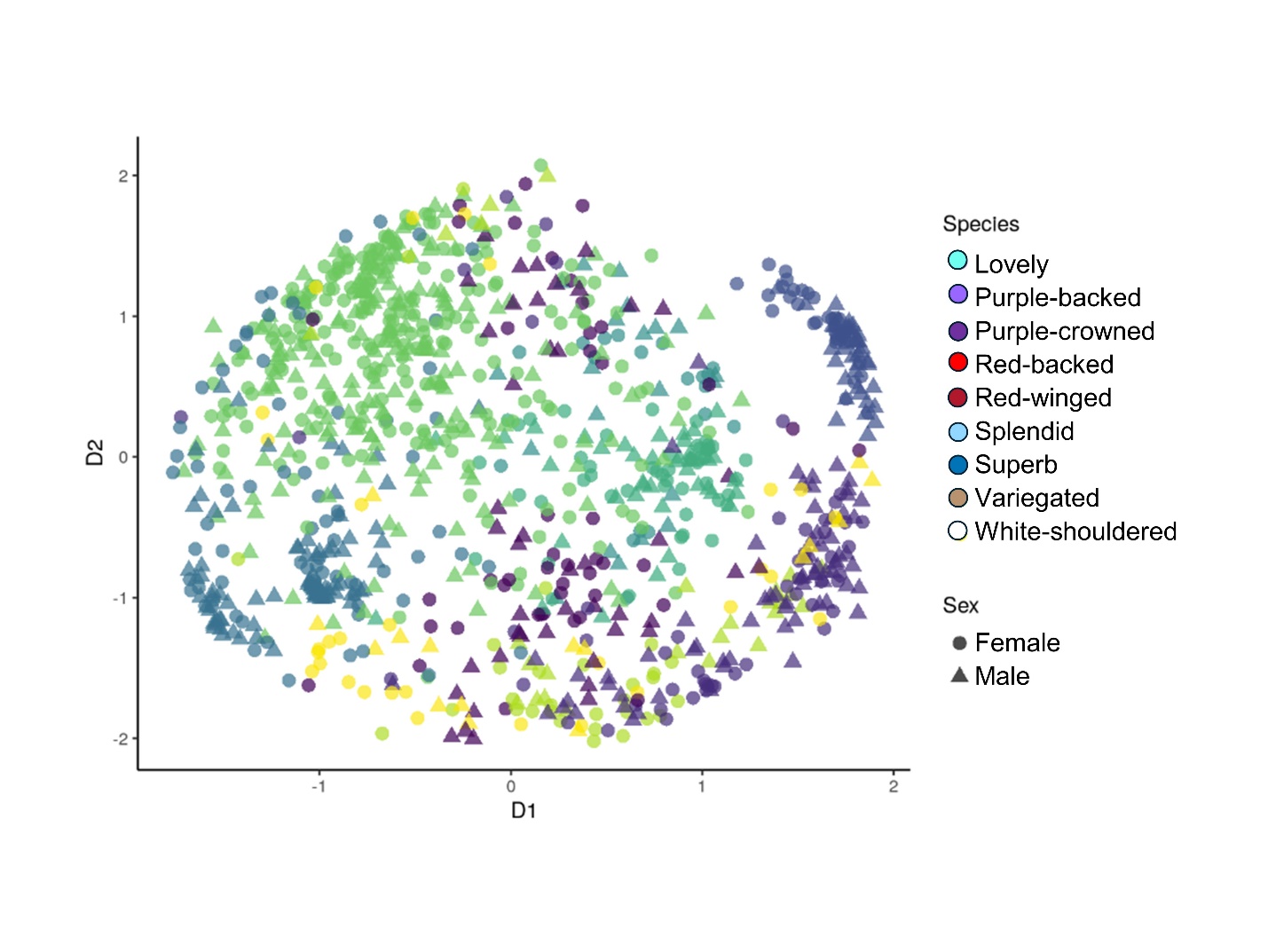


Figure S2


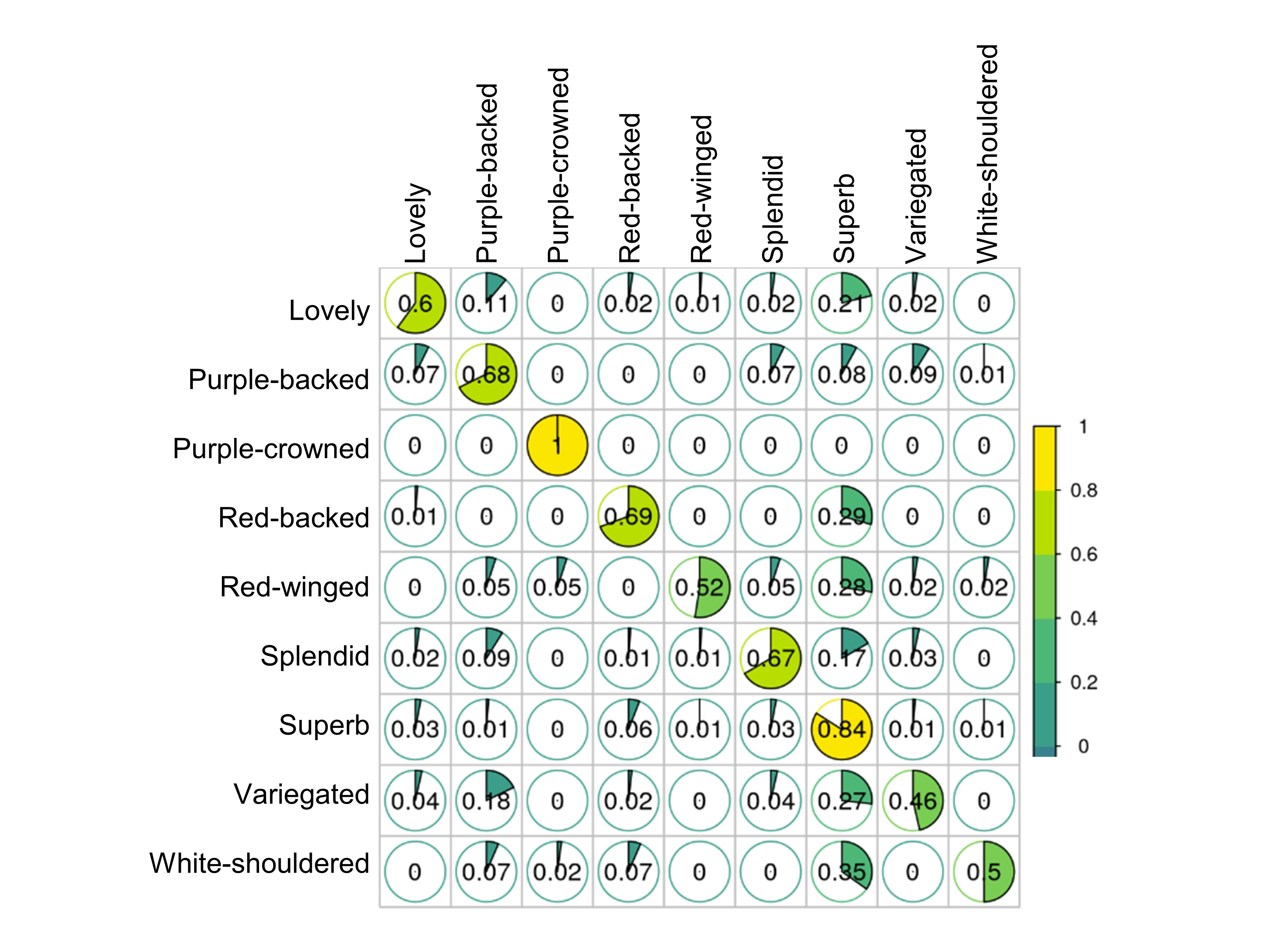


Figure S3


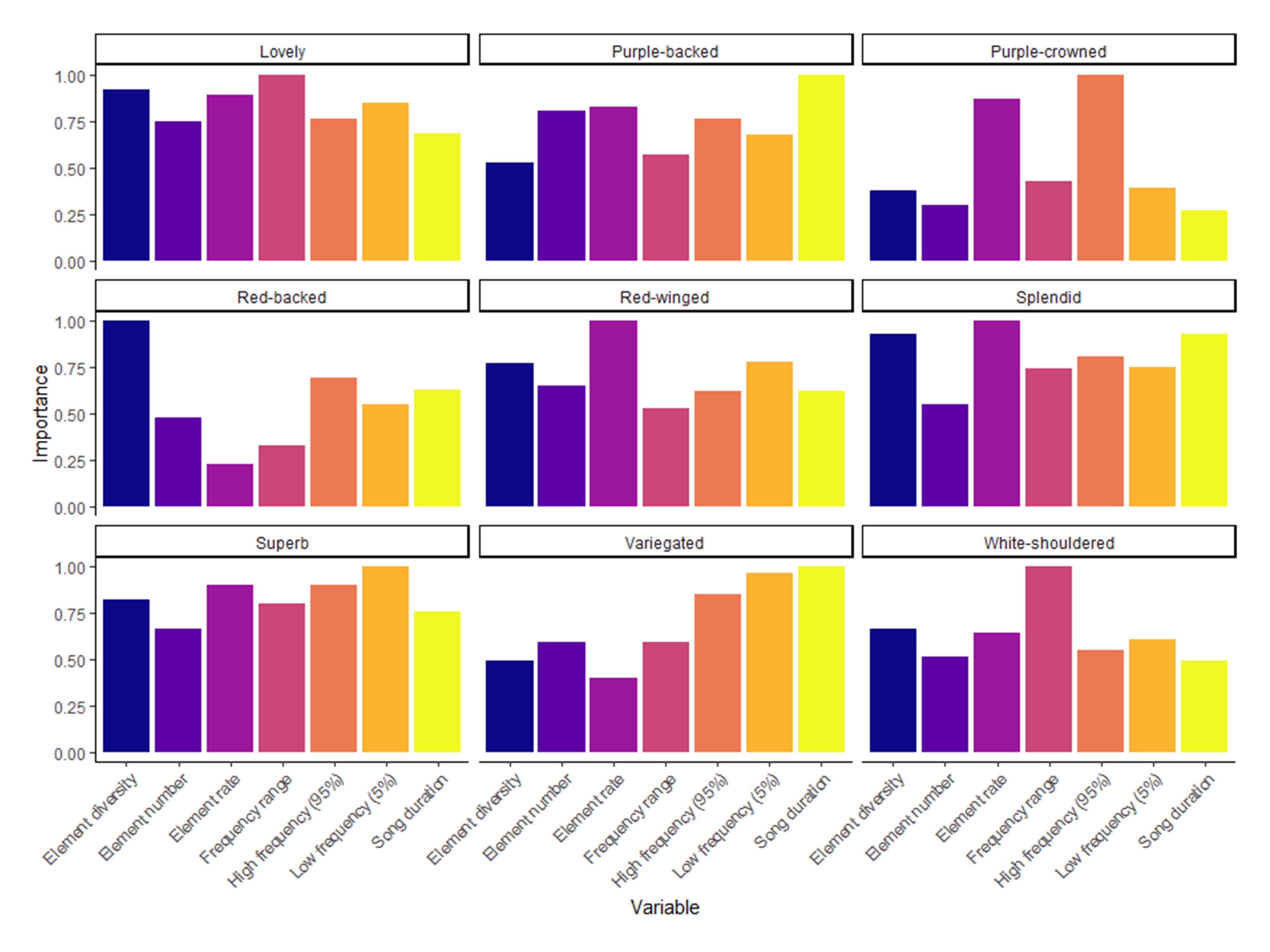


Figure S4

**S5. Model diagnostics.** MCMCglmm model diagnostics for the final evolutionary correlated models, as determined by model selection. To ensure results are robust, all diagnostics were run and compared based on three independent chains. Results for only a single chain are reported in the manuscript.

A. Principle component scores to evaluate evolutionary predictors of male and female fairy-wren song elaboration

1. Gelman–Rubin convergence diagnostic

PC1


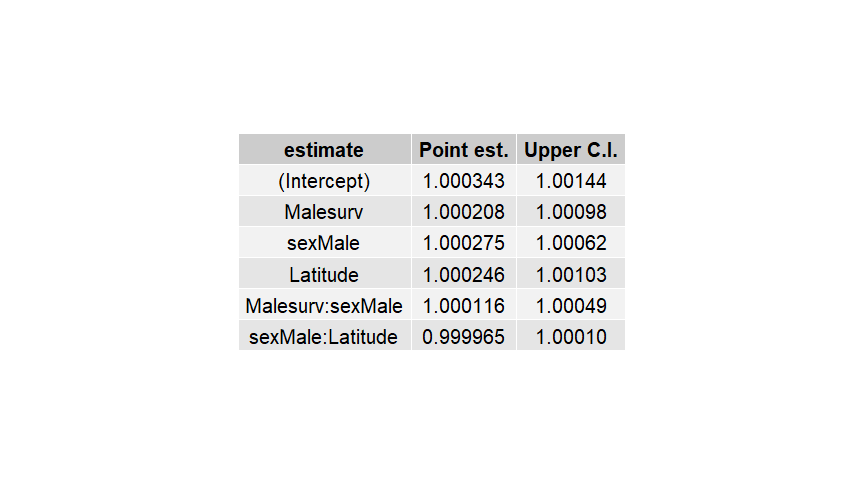


PC2


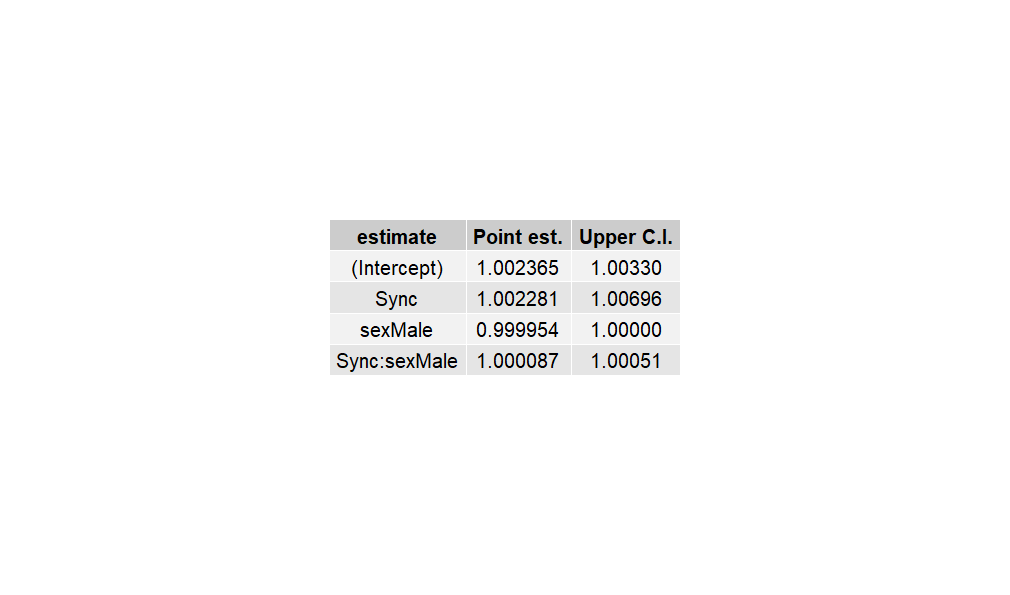


PC3


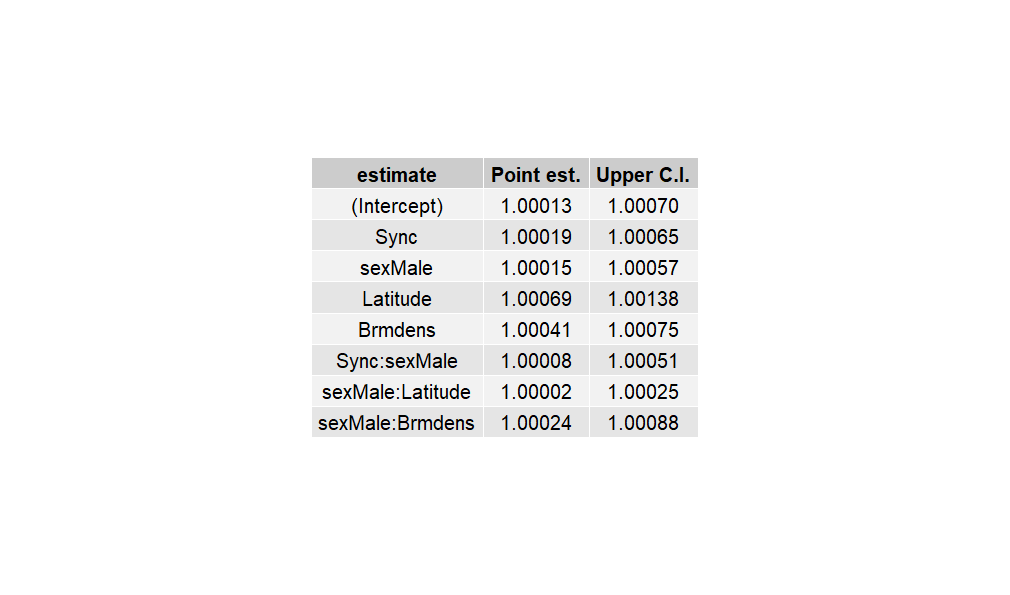


1. Trace plots and autocorrelation plots

PC1


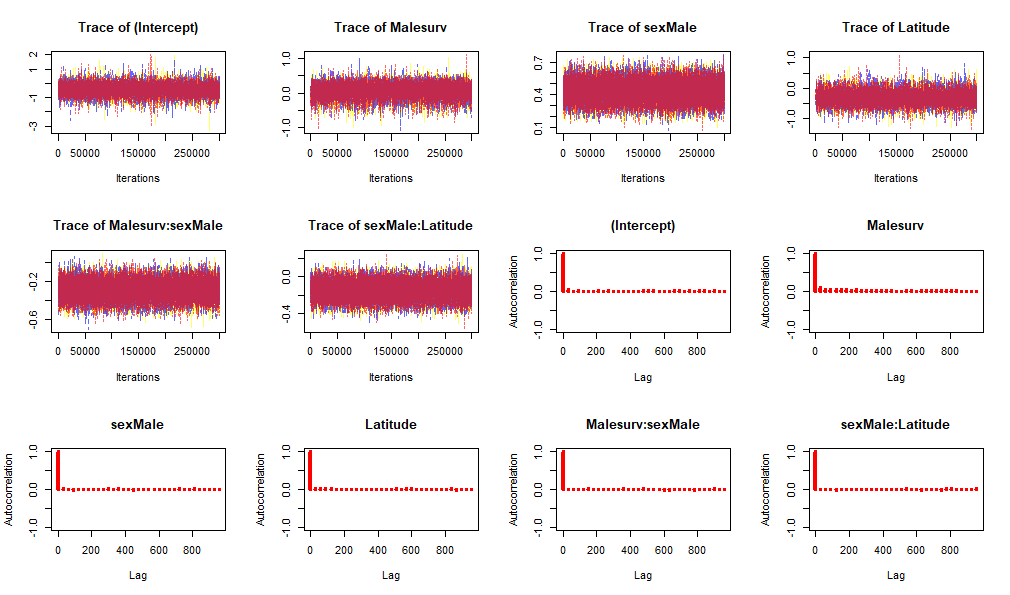


PC2


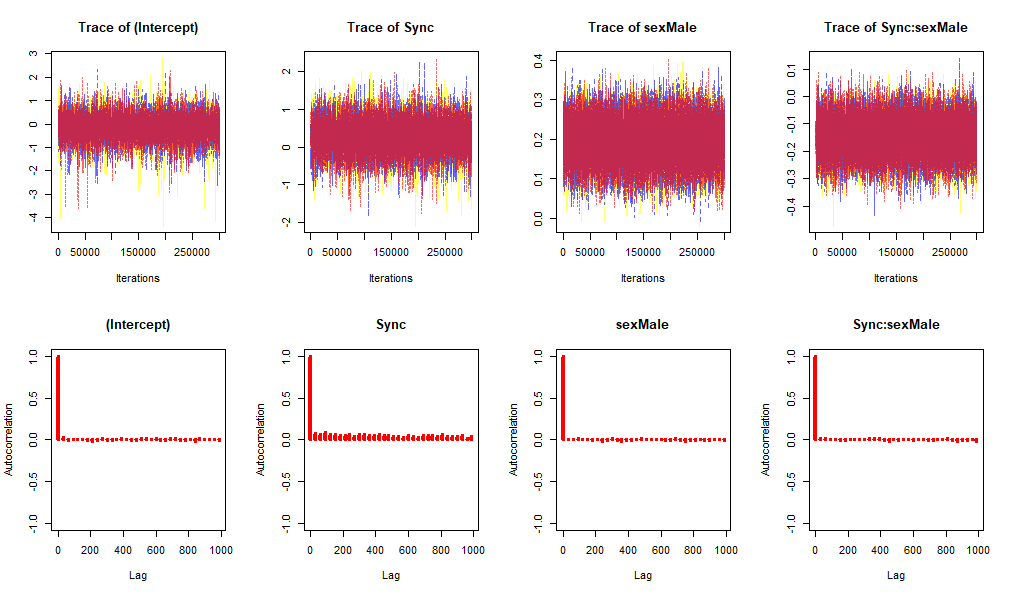


PC3


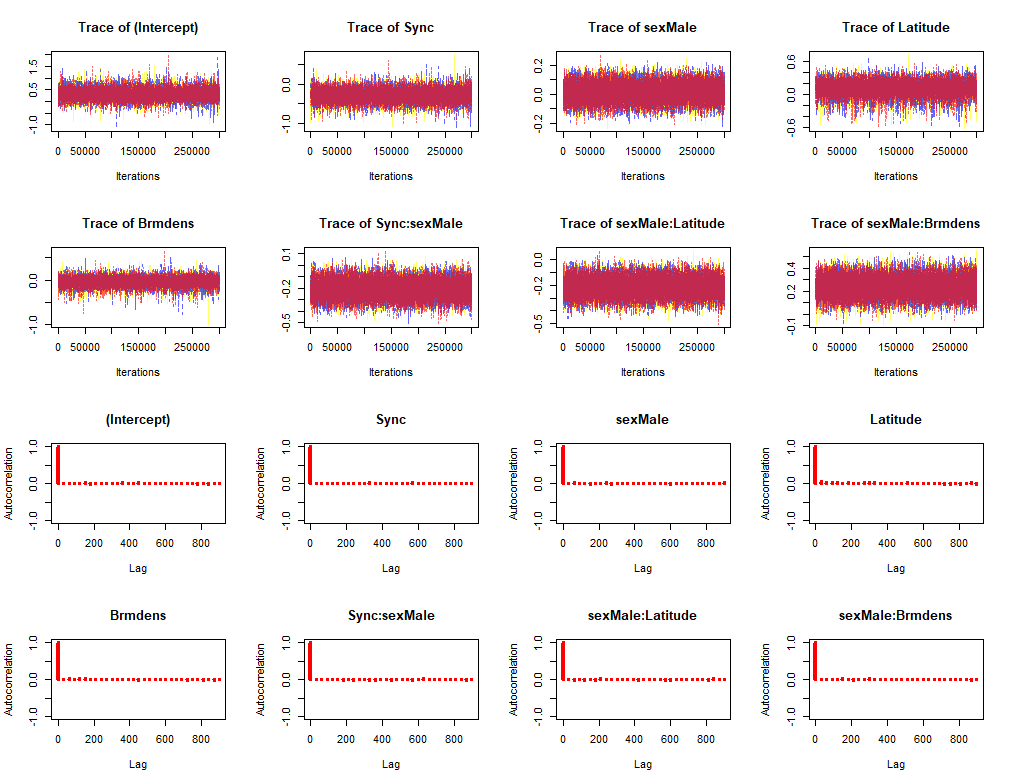


B. Dimorphism metrics to evaluate evolutionary predictors of male and female fairy-wren song dimorphism

1. Gelman–Rubin convergence diagnostics

Area overlap


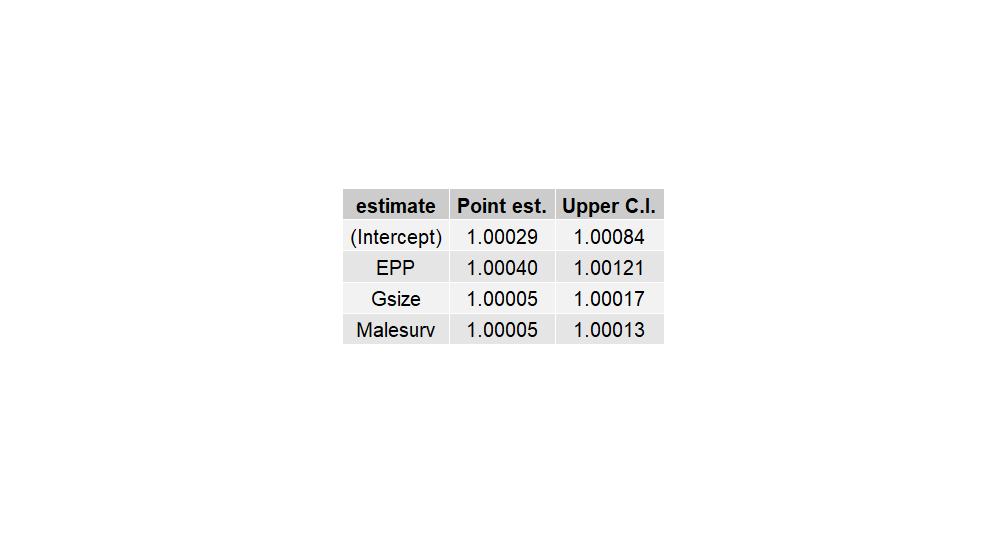


Area distance


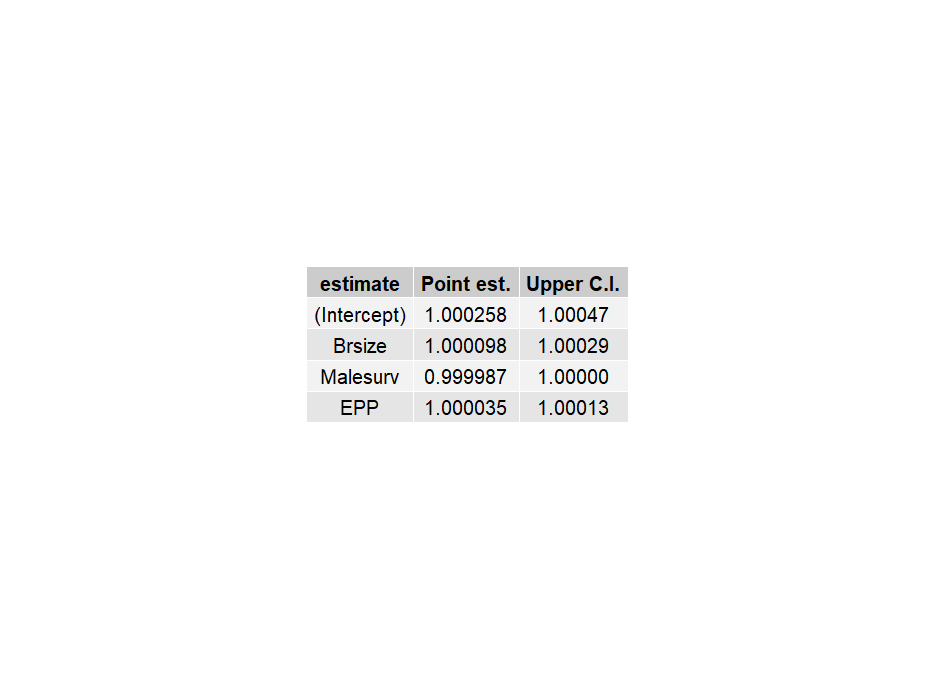


Area size difference


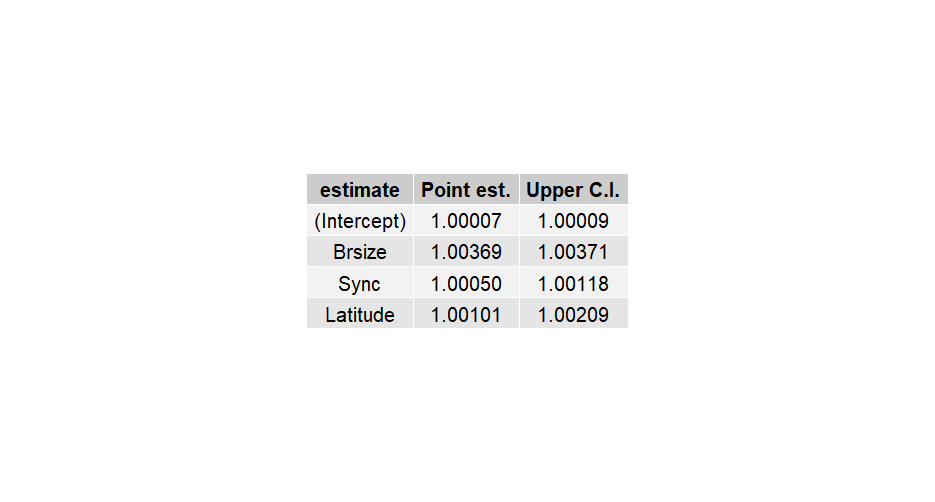


1. Trace plots and autocorrelation plots

Area overlap


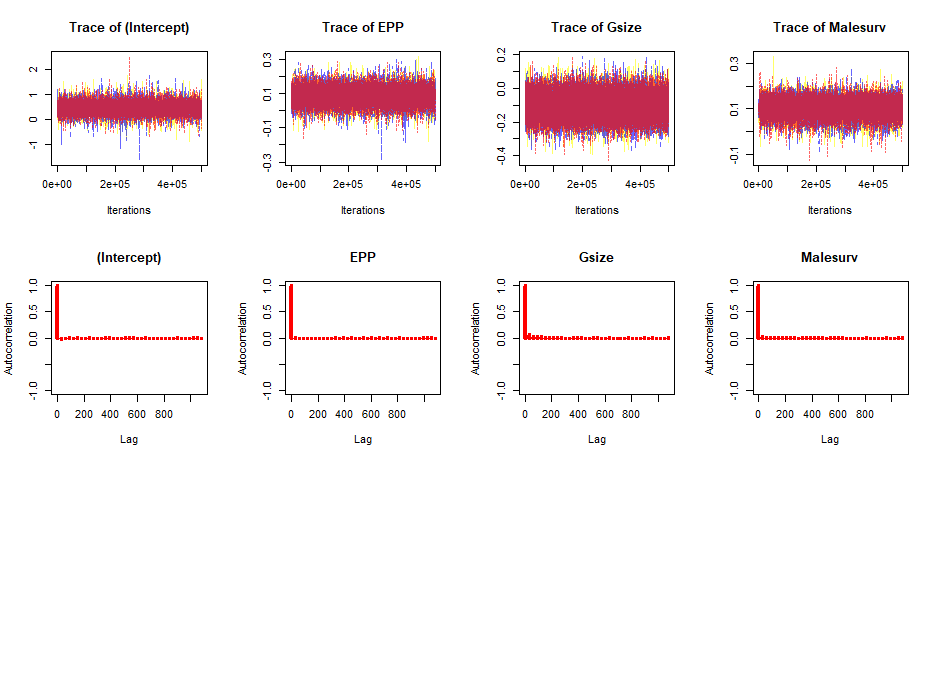


Area distance


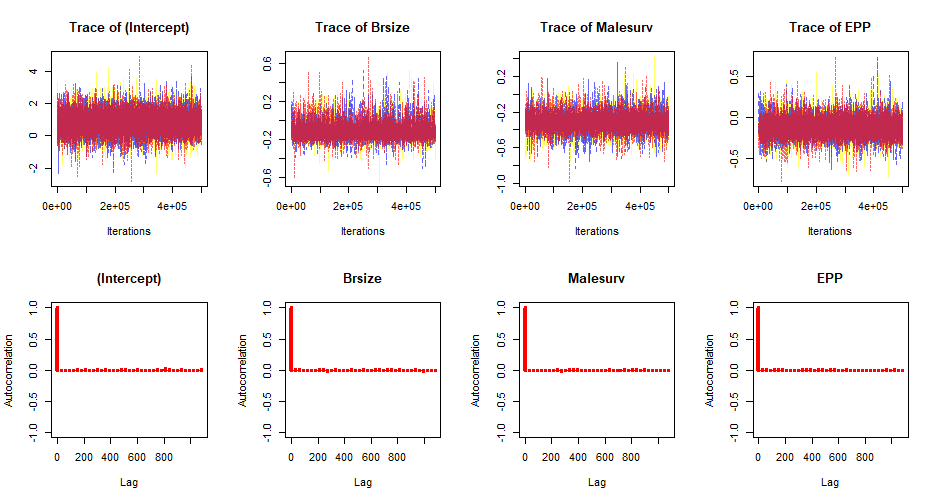


Area size difference


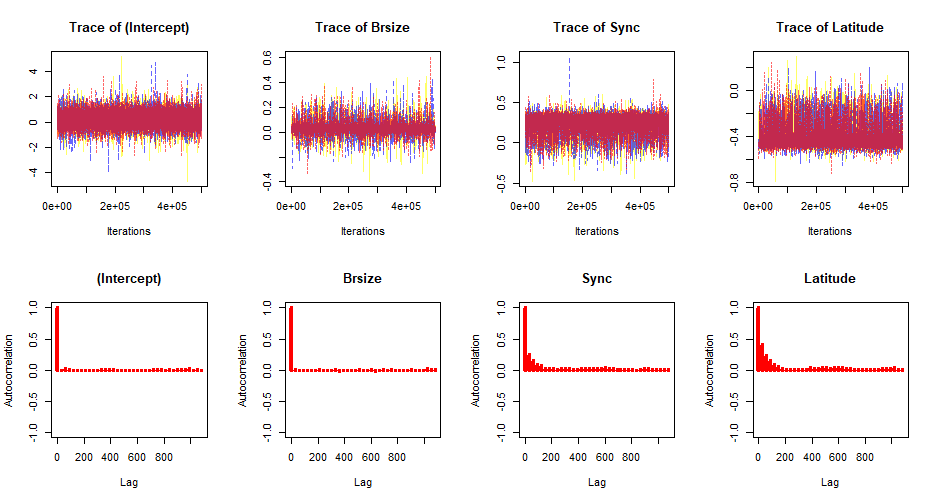

Supplement: Supplementary file 1 — Fig S1‐S5 [file ECE3-11-17901-s002.docx]
